# Metabolic modeling: the link between AI and multi-omics data to accelerate the discovery of antibiotic-resistant traits in ESKAPE pathogens

**DOI:** 10.1128/iai.00435-26

**Published:** 2026-10-07

**Authors:** Nazmul Muzahid, Arjun Patel, Mason Raymond, Tiana Egidi, Nhi Nguyen, Cristal Zuniga

**Affiliations:** 1 Department of Biology, San Diego State University 115105, San Diego, California, USA; 2 DOE Great Lakes Bioenergy Research Center, San Diego State University 7117, San Diego, California, USA; University of California Merced, Merced, California, USA

**Keywords:** computational biology, genome-scale metabolic modeling, flux-to-AI, ESKAPE pathogens

## Abstract

The development of predictive understanding of bacterial resistance to antibiotics is one of the greatest challenges of modern medicine. Genome-scale metabolic models (GEMs) are mathematical representations of the metabolic network of a given organism assembled from genomic data that predict cellular activity across many environmental and genetic contexts. Because a single model can be solved under thousands of conditions, GEMs generate data at scales that support downstream analysis with artificial intelligence tools, helping to elucidate how pathogens adapt to host environments, evade immune responses, and acquire resistance. Here, we review how GEMs of ESKAPE pathogens are built, what data they require, and what they can and cannot do. We describe the reconstruction workflow from automated draft network through manual curation, gap filling, and experimental validation and the public and experimental data sources each stage depends on. We then describe how accessory genes and transcriptomic measurements are incorporated to yield strain- and condition-specific models and where machine learning contributes both in expanding functional annotation and in consuming model-generated flux distributions. Applications are illustrated with ESKAPE examples throughout, including conserved metabolic vulnerabilities identified across strain collections and flux rewiring observed under antibiotic exposure. Despite this progress, high-quality GEMs exist for only a fraction of ESKAPE species; many are constrained by incomplete annotation or limited experimental validation, and non-metabolic resistance mechanisms are captured only insofar as they impose a metabolic cost. Providing reliable tools to build new models from trustworthy templates, therefore, remains a priority for narrowing the gap between genotype and phenotype.

## INTRODUCTION

The alarming prevalence of multidrug-resistant bacteria (MDR; bacteria resistant to three or more classes of antibiotics) poses a serious threat to human health and is worsening. For example, ESKAPE pathogens, including *Enterococcus faecium*, *Staphylococcus aureus*, *Klebsiella pneumoniae*, *Acinetobacter baumannii*, *Pseudomonas aeruginosa*, and *Enterobacter* spp. account for most nosocomial infections, including bloodstream infections, pneumonia, meningitis, urinary tract infections (UTIs), and wounds (1–3).

Infections caused by the ESKAPE pathogens are the leading cause of mortality worldwide, resulting in increased health care costs (4, 5). According to the U.S. Centers for Disease Control and Prevention (CDC)’s 2019 Antimicrobial Resistance Threats Report, over 2.8 million antimicrobial-resistant infections (~2% fatalities) occur in the U.S. each year, incurring healthcare costs of over $4.7 billion (6, 7). Similar statistics have been observed in Europe, with more than 33,000 fatalities that cost $1.5 billion in direct and indirect expenses (8, 9). According to studies, MDR will cause over 24 million individuals to fall below the poverty line by 2030, especially in low-income nations (10). The World Health Organization (WHO) published a list in February 2017 of pathogens for which new experimental- and model-driven antimicrobial strategies are urgently required (11).

By tracking genomic information in ESKAPE, genetic mutations and the acquisition of mobile genetic elements (MGEs) have been observed and linked to AMR (12). Interestingly, ESKAPE pathogens actively adapt and develop resistance to most antibiotics, including last-resort carbapenems and colistin. Between 2020 and 2025, the U.S. Food and Drug Administration (FDA) approved 17 new antibacterial agents, including Exblifep and Orlynvah indicated for complicated and uncomplicated urinary tract infections, respectively (13). These approvals have not offset the long-term slowdown in antibacterial discovery, and most represent modifications of established antibiotic classes rather than agents acting on new targets (13).

By enabling *in silico* prediction of metabolic fluxes and gene essentiality, genome-scale metabolic models (GEMs) provide a mechanistic framework for identifying bottlenecks critical to pathogen survival under both disease and treatment conditions, allowing cellular activity to be evaluated across thousands of environmental contexts (14). Although the clinical management of ESKAPE infections is well established, our understanding of their biology at the system level remains limited. The Big Data era has enabled in-depth characterization of genomic and expression profiles under diverse scenarios through the development of modeling, artificial intelligence, and machine learning (AI/ML) tools (15), and mathematical networks built from these data can clarify biomolecular cross-talk and its functional correlations (16–19). Systems biology tools, such as GEMs, provide predictive strategies for investigating bacterial physiology and discovering new or optimized treatment options based on metabolite cocktails (20, 21). In ESKAPE pathogens, these predictions can be used to identify conserved metabolic vulnerabilities and resistance-associated dependencies and interpret how metabolism shapes host-pathogen interactions and immune responses (20, 22).

Model simulations also bridge the gap between experimental findings and AI/ML algorithms. Because a single GEM can be solved under thousands of simulated genetic and environmental conditions, flux sampling and related methods generate large, internally consistent data sets describing how the network responds to perturbation. These flux distributions act as proxies for experimentally determined expression data, which remain sparse for most ESKAPE strains, and can serve as feature datasets for supervised or unsupervised learning. Hidden patterns at the reaction, metabolite, and gene level that are not apparent from the genome sequence alone can then be revealed (23, 24).

The foundations for the continuous development of predictive biological understanding are critical for narrowing the gap between genotype and phenotype and guiding appropriate interventions. Despite progress, high-quality GEMs are only available for a fraction of ESKAPE species, and many models are constrained by insufficient annotations or a lack of experimental validation. Expansion of these models will facilitate the translation of academic research into therapeutic applications to address AMR in the twenty-first century. This review aims to provide up-to-date information on GEM and AI/ML in ESKAPE pathogens, with a focus on current and emerging drug development avenues to understand, control, and respond to AMR.

## PREVALENCE AND CLINICAL IMPACT OF AMR IN ESKAPE PATHOGENS

In high-risk settings, such as intensive care units, ESKAPE pathogens account for over 50% of hospital-acquired infections (25, 26), and in a U.S. multicenter analysis of more than 1.1 million patient encounters, they represent 42.2% of bloodstream infection isolates, ranging regionally from 39.5 to 49.9% (27). Reported rates are higher in some settings; ESKAPE organisms accounted for 73–76% of nosocomial isolates in Romanian and Turkish intensive care units surveyed between 2022 and 2024 (25, 28). Compared with patients infected by non-ESKAPE organisms, patients with ESKAPE infections remained hospitalized longer (median 1.0 day, mean 3.3 days) and incurred higher costs of care (median $2,600, mean $5,500) (27). The same analysis reported a 2.1% absolute increase in all-cause mortality at discharge (8.7% versus 6.6%) (27, 29), and 30-day mortality of 43.3% has been reported for ESKAPE infections overall, rising to 48.2% among MDR cases (30). Reported 30-day mortality varies substantially by species (Table 1). Different studies suggest that AMR could cost the global economy between $300 billion and $1 trillion annually by 2050 if no action is taken (31).

**TABLE 1:** Clinical impact of ESKAPE pathogens in human infections

ESKAPE species	Clinical manifestations	Mortality rates
*Enterococcus faecium*	Clusters in UTI, intra-abdominal infection, bacteremia, and endocarditis (32)	30-Day enterococcal bloodstream infections (BSI) mortality 20–35% (33)
*Staphylococcus aureus*	Skin/soft-tissue infections (cellulitis, abscesses, impetigo) (34), bacteremia, endocarditis, osteoarticular disease, and toxin syndromes, septic arthritis, pneumonia (35)	15–31% 30-day mortality for *S. aureus* bacteremia (36)
*Klebsiella pneumoniae*	Hospital-acquired pneumonia (HAP), ventilator-associated pneumonia (VAP), meningitis, UTI, bacteremia, pyogenic liver abscesses (37, 38)	Meta-analysis of 157 studies: pooled 30-day mortality 29% (39)
*Acinetobacter baumannii*	HAP, VAP, bloodstream, skin, and urinary tract infections, and secondary meningitis, catheter-associated UTI (40, 41)	*A. baumannii* BSI 34% 30-day mortality (higher with CRAB/ICU) (42)
*Pseudomonas aeruginosa*	HAP, VAP, chronic airway infection in cystic fibrosis/bronchiectasis, bacteremia, UTI, skin and soft tissue infection (43)	22.5% 30-day mortality (higher in ICU/oncology series) (44)
*Enterobacter* spp.	UTI, bacteremia, neonatal pneumonia, skin and soft tissue infection, intra-abdominal infection, endocarditis, septic arthritis (32)	19.6% 30-day mortality for Enterobacter bacteremia (45)

These figures carry an important caveat. Global burden estimates report two distinct quantities: deaths *attributable to* resistance, where the counterfactual is the same infection caused by a susceptible organism, and deaths *associated with* resistance, where the counterfactual is no infection at all. For 2019, these definitions differed roughly fourfold at 1.27 million and 4.95 million deaths, respectively (46). The figure of approximately 1.3 million deaths commonly cited for AMR, around 80% of which involve ESKAPE pathogens (46), is the narrower attributable estimate. The choice of counterfactual is not a technical detail; it determines what the number means and which interventions it justifies (47). Most single-center studies instead report crude mortality among patients harboring a resistant organism, a design that cannot distinguish death caused by resistance from death in a patient who happens to carry a resistant organism (48). Because resistant infections concentrate in patients who are already critically ill, and because case-control studies matching patients on otherwise identical clinical condition are extremely difficult to assemble, reported mortality differentials are likely confounded by underlying illness severity (47, 49). The consistency of these associations across settings supports a real burden, but individual estimates should be read as upper bounds.

Surveys of fecal carriage among non-hospitalized community members detected *A. baumannii* in 5% of individuals sampled and *Escherichia coli* in 17.82% (40, 50). *E. coli* is not part of the ESKAPE acronym as originally defined but is frequently included as a second “E” in the expanded ESKAPEE grouping since it shares resistance determinants and clinical niches with the six ESKAPE species (40, 50, 51). The data from a literature review focused on the prevalence of ESKAPE pathogens in Africa revealed that the total prevalence of these pathogens in humans, animals, the environment (water, soil, and surfaces), and food sources was 35.8, 37.3, 47.7, and 34.2%, respectively (34). Table 1 shows the clinical manifestations and mortality rates of ESKAPE pathogens.

Although most studies on ESKAPE pathogens have focused on healthcare facilities, these strains have also been found in non-hospital settings. They have been identified in a variety of environmental sources, including soil, food (vegetables, fruit, and animal products), various water sources (surface runoff, drinking water, wastewater, streams, dams, and rivers), plants, and sewage or municipal waste (4, 52–54). Studies suggest that antibiotic-resistant ESKAPE strains circulate in these settings, acting as reservoirs and facilitating transmission to people and animals (34).

Resistance in these organisms arises through both intrinsic mechanisms encoded on the chromosome and acquired mechanisms driven by mutation under selective pressure or by horizontal transfer of mobile genetic elements (55). Six mechanisms recur across the group: enzymatic inactivation of the drug, modification of the drug binding site, reduced outer membrane permeability, active efflux, biofilm formation, and horizontal transfer of resistance determinants (32, 55–65). These mechanisms are well described elsewhere and not reviewed in detail here; what remains poorly resolved is how they are coordinated with the metabolic state of the cell, which is the problem genome-scale metabolic modeling is positioned to address.

## GENOMIC DATA AND THE CHALLENGE OF UNKNOWN GENE FUNCTION IN ESKAPE

The “unknownome” refers to the large set of genes and gene products whose biological functions are not yet known or are only poorly characterized. It includes genes annotated as “hypothetical proteins” or as having vague labels, such as “function unknown.” The unknownome exists in all genomes, and, in *E. coli*, it is also referred to as the Y-ome (66). A substantial fraction of genes in every ESKAPE genome falls into this category. Even in *E. coli*, the most intensively studied bacterium, 35% of genes still lack direct experimental evidence of function (66), and annotation quality for ESKAPE species is generally poorer. In *Enterobacter*, a pangenome of 777 complete genomes found genes of unknown function to be the single largest category of the accessory genome at 48%, accounting for a further 21% of core genes and 68% of the rare genome (67). These are more accurately described as genes of unknown function than as genes of no consequence. Systematic phenotyping of mutant libraries across dozens of bacterial species has assigned specific growth and stress phenotypes to thousands of previously uncharacterized genes (68), and targeted screens in staphylococci have implicated uncharacterized loci in antibiotic susceptibility and biofilm formation (69). In *P. aeruginosa*, structure-based characterization of proteins of unknown function has been used to nominate candidate drug targets (70). Genes of unknown function, therefore, represent both the largest gap in ESKAPE annotation and an underexploited source of intervention points.

Several complementary strategies exist for assigning function to these genes. Randomly barcoded transposon sequencing generates fitness profiles for entire mutant libraries across hundreds of conditions, allowing uncharacterized genes to be placed into pathways by phenotypic similarity to genes of known function (68). CRISPR interference extends this to essential genes, which cannot be deleted outright, and pairs knockdown with transcriptomic or imaging readouts (69). Where mutant libraries are unavailable, guilt-by-association approaches infer function computationally, clustering genes by co-expression across large transcriptomic compendia and applying machine learning to predict functional categories (71). Metabolic modeling contributes a distinct signal: when a curated network requires a reaction for which no gene has been assigned, the model specifies the biochemical function that must exist, converting an open-ended annotation problem into a targeted search. This model-enabled gene search strategy has directly identified enzymatic and transport functions that homology-based annotation had missed (72). Applied systematically to ESKAPE genomes, these approaches offer a route to shrinking the unknownome rather than cataloging it.

Thousands of genomes from ESKAPE pathogens have been fully sequenced and curated in databases, such as National Center for Biotechnology Information (NCBI), PubMLST, and other public repositories (12, 73, 74). Those data sets are massive and continue to grow. Besides, short- (Illumina) and long-read sequencing (Oxford Nanopore Technologies, PacBio) are now routine in surveillance and research using tools, such as multilocus sequence typing (MLST), cgMLST, and single nucleotide polymorphism (SNP)-based phylogenomics (75, 76). Long-read sequencing helps obtain complete genome sequences, resolve plasmids, genomic islands, and structural variants, and is beginning to map methylomes (bacterial epigenetics) (75, 77, 78).

Public databases do not provide diverse or high-quality genomes for all taxa, particularly for less prevalent species and strains, even where substantial sequencing has been performed (79). Genome quality is a particular problem for mobile genetic elements. Short-read assemblies fragment repetitive regions, and plasmids are frequently split across contigs or lost entirely, with small and cryptic plasmids recovered least reliably (80, 81). Long-read and hybrid assembly substantially improve plasmid resolution in clinical isolates of *E. coli* and *K. pneumoniae* (82), but most publicly deposited ESKAPE genomes predate routine long-read sequencing. This matters directly for modeling because resistance determinants are disproportionately plasmid-borne (12), so reconstructions built from fragmented assemblies will systematically under-represent accessory metabolic and resistance functions. Beyond assembly quality, challenges remain in the functional annotation of resistance, pathogenicity, and host adaptability, along with inconsistent metadata and reporting standards across clinical, community, environmental, and animal isolates (83).

## HOW GENOME-SCALE METABOLIC MODELS ARE BUILT AND WHAT THEY REQUIRE

**Fig 1:**
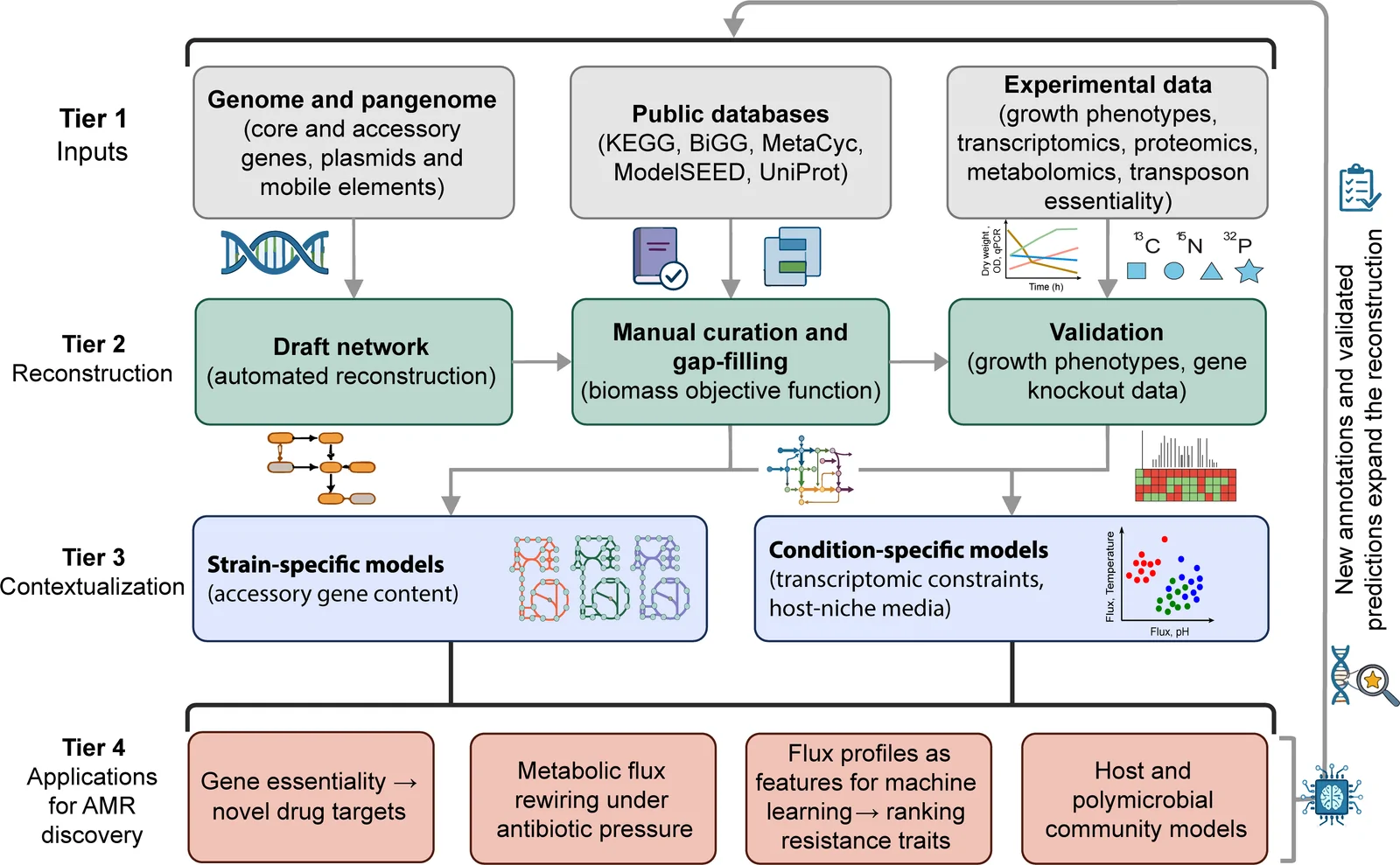
Reconstruction and application of genome-scale metabolic models (GEMs) for ESKAPE pathogens. Tier 1: genome and pangenome sequences supply gene content; public biochemical databases supply reaction and annotation data; and experimental measurements supply the phenotypes used for curation and validation. Tier 2: an automated draft network is manually curated and gap-filled; a biomass objective function is defined; and predictions are tested against growth and gene knockout data. Machine learning contributes at this stage by expanding functional annotation of genes left uncharacterized by homology-based methods. Tier 3: curated models are specialized to individual strains using accessory gene content or to individual conditions using transcriptomic constraints and host-niche media. Tier 4: both classes of contextualized models support all four applications shown, as indicated by the horizontal connector spanning the row. Machine learning contributes a second time here, consuming model-generated flux distributions as feature sets for ranking resistance-associated traits. The feedback arrow indicates that new annotations and validated predictions return to the reconstruction.

A genome-scale metabolic model is a mathematical representation of every metabolic reaction an organism is predicted to catalyze, linked to the genes encoding the responsible enzymes. Reconstruction proceeds in four stages (Fig. 1). A draft network is generated automatically by mapping annotated genes onto reactions in a biochemical reference database, a step that tools, such as CarveMe, gapseq, and ModelSEED, complete in minutes (84–86). The draft is then manually curated: reactions are checked against the primary literature; gene-protein-reaction associations are corrected; and a biomass objective function is defined, specifying the metabolites the organism must produce in order to replicate (87). Gaps that prevent biomass production are closed by adding the minimum set of reactions required, a step that generates explicit hypotheses about missing enzyme functions. Finally, predictions are compared against experimental phenotypes, most often growth on defined carbon and nitrogen sources and gene essentiality measured by transposon or knockout screens (88). Standardized tests are now available to assess reconstruction completeness and internal consistency before a model isused (89).

The data a reconstruction requires fall into two categories. The network itself is assembled almost entirely from public resources: genome annotations from NCBI, and reaction, enzyme, and pathway content from curated databases, such as Kyoto Encyclopedia of Genes and Genomes (KEGG), MetaCyc, BiGG, and ModelSEED (86, 90). No organism-specific experimental characterization of individual genes is needed to produce a draft. The experimental data that are needed measure phenotype rather than gene function: growth or no growth on defined media, substrate uptake and product secretion rates, and essentiality screens, all of which are used to test and correct the network rather than to build it. A usable reconstruction can, therefore, be produced for any sequenced ESKAPE isolate, but its reliability is bounded by the quality of the underlying annotation and by how much phenotypic data exist to validate it against. This is why reconstruction coverage across the ESKAPE group is uneven, and why models for the better-characterized species can be trusted further than those for the rest.

Because resistance and virulence determinants are frequently accessory rather than core, a single reference model cannot represent a species. Strain-specific reconstruction addresses this by mapping each strain’s gene complement onto a curated species-level template, retaining reactions supported by genes the strain carries and removing those it lacks. The first such application in an ESKAPE pathogen covered 64 *Staphylococcus aureus* strains and recovered strain-specific metabolic capabilities associated with pathogenic behavior (91). Comparable approaches have been applied to *Klebsiella pneumoniae* both in focused comparisons of resistant clinical isolates (92) and through automated pipelines that generate strain-specific models across hundreds of genomes (93). The resulting models render the accessory genome interpretable in metabolic terms, converting gene presence and absence into predicted differences in nutrient utilization, growth capability, and gene essentiality.

Transcriptomic data enter as constraints rather than as inputs to network structure. A curated model describes what an organism can do; expression data indicate what it is doing under a particular condition. Integration algorithms use transcript abundance to restrict flux through reactions whose genes are poorly expressed and permit flux through those that are highly expressed, yielding a condition-specific model (94–96). Applied to an ESKAPE isolate under antibiotic exposure, this produces a prediction of how metabolic flux is redistributed during that exposure, which the expression data alone do not provide. These algorithms differ substantially in their assumptions and in the predictions they generate, and benchmarking shows that none is uniformly best (97), so the choice of integration method should be reported and justified rather than treated as a technical detail.

Reconstruction also operates in the reverse direction using network structure to inform annotation. When gap-filling establishes that a reaction must be present for an organism to grow as observed, but no gene has been assigned to it, the model has specified a biochemical function that the genome must encode somewhere. This converts an open-ended annotation problem into a targeted search across candidate genes and has been used to assign enzymatic and transport functions that homology-based methods had missed (72). For ESKAPE species, where a large fraction of each genome remains functionally uncharacterized, this is one of the few systematic routes to reducing the unknownome rather than simply describing it.

Machine learning enters this workflow at two distinct points. Upstream, it improves the annotation on which reconstruction depends, predicting functional categories for uncharacterized genes from expression patterns, sequence embeddings, and structural features (71). Downstream, it consumes what models produce: because a single reconstruction can be solved under thousands of simulated genetic and environmental conditions, it generates large, internally consistent flux data sets that serve as feature sets for classification, including for phenotypes, such as resistance, where experimental training data are scarce (23). Machine learning is, therefore, not an alternative to mechanistic modeling in this context but a complement to it, addressing the annotation bottleneck at one end of the workflow and the data-volume bottleneck at the other.

The advantage of this framework for resistance research is that it makes explicit, testable predictions about which metabolic functions an organism requires under a defined condition. Gene essentiality predictions nominate candidate drug targets, as they have for *S. aureus*, for colistin-resistant *Acinetobacter baumannii*, and for *Pseudomonas aeruginosa* biofilms (16, 98, 99), and simulated antibiotic exposure predicts the flux rewiring that accompanies adaptation (100). The limitations are equally concrete. Reconstructions represent metabolism alone, so resistance arising through target modification, efflux, or enzymatic inactivation is captured only insofar as it imposes a metabolic cost. Flux balance analysis assumes a metabolic steady state and an objective function, usually growth, which may describe neither a persister cell nor a mature biofilm. Predictions inherit every error present in the underlying annotation. Model quality across ESKAPE remains uneven, with several species represented only by automatically generated reconstructions that have not been manually curated or experimentally validated. Genome-scale models are best understood as a framework for generating prioritized, mechanistically grounded hypotheses about metabolic vulnerability, not as a substitute for experimental confirmation.

## GENOME-SCALE METABOLIC MODELING OF ESKAPE PATHOGENS

Applied to ESKAPE pathogens, reconstructions have revealed broad metabolic plasticity and the ability to exploit diverse carbon, nitrogen, and micronutrient sources across host niches, a property that underpins persistence while also exposing conserved biosynthetic vulnerabilities amenable to drug targeting (101). Combining these models with large-scale omics data has made metabolic rewiring under antibiotic pressure directly observable: integrating transcriptomic and metabolomic measurements with a genome-scale model of *A. baumannii* defined the metabolic response to polymyxin treatment (102), and expression of the KPC-2 carbapenemase in *K. pneumoniae* drives systemic flux redistribution beyond the resistance enzyme itself (100). Contextualizing clinical isolate evolution data has likewise supported identification of efflux systems of the MFS, RND, and ABC families as intervention targets through robustness analysis and regulatory network frameworks (103, 104). Other studies have focused on proteomics data, such as matrix-assisted laser desorption/ionization-time of flight (MALDI-TOF) mass spectrometry, enabling rapid identification of resistant strains (105), while multi-omics antigen discovery informs monoclonal antibody development against conserved epitopes (106).

**Fig 2:**
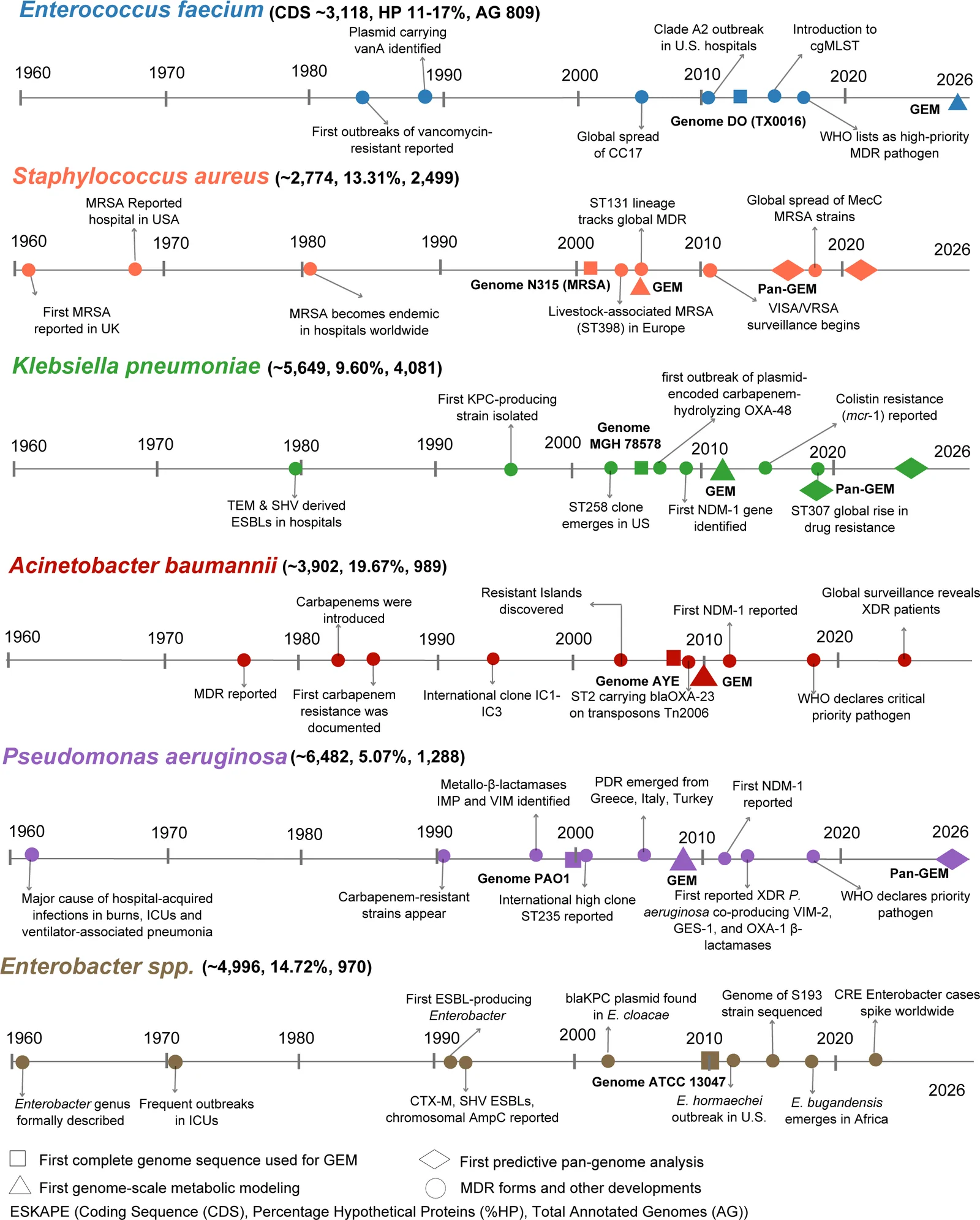
Timeline of genomic and modeling events in ESKAPE pathogens. Next to each ESKAPE name are the average coding sequence length (CDS) calculated from the complete genome sequences present in BV-BRC (https://www.bv-brc.org/), the percentage of hypothetical proteins (HP) calculated from the NCBI reference genome, and the total annotated genome content (AG). Circles mark antimicrobial resistance, genomic, and epidemiological milestones, including first reports of resistant phenotypes and resistance determinants, emergence and spread of dominant clones, genome sequencing of additional strains, developments in surveillance and typing, and WHO priority designations. Squares mark the publication year of the genome sequence of the strain on which that organism’s curated genome-scale metabolic model was built, with the strain named in each case; for *Enterobacter*, for which no curated model has been reported, the square marks the first published complete genome of the type species. Triangles mark the year the first manually curated genome-scale metabolic model became available, and, diamonds, the year of the first predictive pangenome analysis defined here as a pangenome coupled to strain-specific metabolic models. Tables listing every plotted event with its source are provided in the Supplemental material.

Decades typically separate the first description of an ESKAPE species from its first genome sequence and longer still from its first genome-scale reconstruction. *Enterococcus faecium*, for example, was first isolated in 1919, although it was initially classified within the genus *Streptococcus* and not transferred to *Enterococcus* until 1984 (107). Reconstruction coverage remains uneven across the group: the first curated *Enterococcus* metabolic model was built for *E. faecalis* V583 (108) and applied to identify critical autotrophies for growth. As computational power and omics data sets expand, GEMs are increasingly positioned as dynamic, system-level tools for precision antimicrobial development. Figure 2 highlights the milestones in identification and metabolic modeling achieved since 1960 in ESKAPE pathogen research. For example, *Staphylococcus aureus* is the best-characterized ESKAPE pathogen. Early manually curated reconstructions, including *i*SB619, established the metabolic framework for predicting growth phenotypes and gene essentiality in laboratory strains. These models were subsequently expanded and refined, improving metabolic coverage, reaction annotation, and predictive accuracy. When the models were combined with proteomic and transcriptomic profiling, it was shown that surface protein expression varies with regulatory genotype and adherence phenotype, linking regulatory state to the cell-surface proteins that mediate host interaction (109). For *Klebsiella pneumoniae*, genome-scale metabolic modeling of clinical isolates was published in 2011, highlighting adaptive rewiring of amino-acid catabolism (notably valine utilization) that correlates with clinical lineage and may represent a metabolic liability for therapeutic exploitation (12, 110). A reconstruction of *Acinetobacter baumannii* predicted growth on more than 100 carbon sources with ~85% accuracy, demonstrating adaptability to diverse host niches. The combination of a model with experimental metabolomics also enabled the identification of non-human homologous metabolic genes as candidate antibiotic targets, demonstrating the power of integrated modeling to propose species-specific interventions (111, 112). *Pseudomonas aeruginosa* illustrates how much reconstruction quality matters. Successive PAO1 and PA14 models predict gene essentiality against transposon mutant libraries with accuracies ranging from roughly 65% for early reconstructions to above 90% for curated updates validated across multiple independent screens (113, 114), a spread that reflects depth of curation rather than any property of the organism. Where large transcriptome compendia exist, machine learning has been applied to infer the transcriptional regulatory network and identify condition-specific modules, including quorum sensing, respiration, and exometabolome changes, that determine whether isolates favor growth, biofilm formation, or secretion of virulence metabolites (19). For *Enterobacter* spp., pangenome analysis of 777 complete genomes resolved the genus into lineage-associated and horizontally acquired gene sets, with metabolic genes forming the largest functional category of the core genome and resistance determinants concentrated in the plasmid-borne and rare fractions rather than the core (67, 115). Lineage-restricted metabolic traits are evident in this structure, including a pyrroloquinoline quinone biosynthetic operon confined to *Enterobacter hormaechei* (67), but no curated genome-scale reconstruction has been reported for the genus, so these distinctions have not been translated into metabolic predictions.

## MODEL-DRIVEN PANGENOME ANALYSIS

Pangenomic frameworks distinguish core genes shared across all strains from accessory and unique genes that confer strain-specific traits (116). Coupling these frameworks to metabolic reconstruction converts gene presence and absence into predicted phenotype, and the resulting studies point to a recurring set of pathways of major importance for resistance and survival (Table 2). The first model-driven pangenome of an ESKAPE pathogen covered 64 *S. aureus* strains and recovered strain-specific metabolic capabilities linked to pathogenic behavior (91). This pangenome analysis was published in 2016; 114 published strain-specific models were subsequently curated and compared (Fig. 2). An earlier comparative reconstruction across 13 *S. aureus* strains had already established the pattern that makes the approach useful: of 70 enzymes predicted to be essential in at least one strain, 44 were essential in all of them, and of 54 synthetic-lethal enzyme pairs, only 10 were shared across every strain (98). Six of those 10 fall within amino sugar and peptidoglycan biosynthesis, making cell wall precursor supply the most concentrated vulnerability identified across the species. The contrast between conserved and strain-restricted targets is the practical argument for strain-specific rather than species-level modeling: conserved enzymes are broad-spectrum candidates, while strain-restricted ones explain why a single reference model misrepresents clinical diversity. Conserved two-component systems, such as histidine kinases, vary across strains in the same way, underscoring their role in ecological adaptation (117).

**TABLE 2:** Metabolic pathways and processes identified as points of major importance by genome-scale modeling in ESKAPE pathogens^*a*^

Pathway or process	Organism	Modeling finding	References
Amino sugar and peptidoglycan biosynthesis	*S. aureus*	Six of 10 strain-independent synthetic-lethal enzyme pairs; *murA* and *murB* unconditionally essential	(98)
Fatty acid biosynthesis	*S. aureus*	Eight single essential enzymes, including *fabD*, *fabG*, *fabZ*, and acetyl-CoA carboxylase	(98)
Shikimate and aromatic amino acid biosynthesis	*S. aureus*	*aroA*, *aroB*, *aroC*, *aroE*, and tryptophan pathway enzymes essential in all 13 strains; pathway absent in humans	(98)
Histidine biosynthesis	*S. aureus*	Complete *his* operon essential; pathway absent in humans	(98)
Porphyrin biosynthesis	*S. aureus*	*hemB*, *hemC*, and *hemD* essential across strains	(98)
Amino acid catabolism (valine)	*K. pneumoniae*	Rewiring correlates with clinical lineage; proposed metabolic liability	(12, 110)
Central carbon metabolism under carbapenemase expression	*K. pneumoniae*	KPC-2 expression drives systemic flux redistribution	(100)
Metabolic reprogramming under colistin	*A. baumannii*	Transcriptome-constrained model identifies targets in colistin-exposed cells	(99)
Polymyxin stress response	*A. baumannii*	Transcriptomics and metabolomics integrated with GEM define treatment response	(102)
Biofilm-associated metabolism	*P. aeruginosa*	Targets specific to the biofilm state rather than planktonic growth	(16)
Efflux systems (MFS, RND, ABC)	Multiple	Identified as intervention points by robustness and regulatory network analysis	(103, 104)

^*a*^ *E. faecium* and *Enterobacter* spp. are not represented. No curated reconstruction has been reported for *Enterobacter*, and modeling of *E. faecium* has not yet identified resistance-associated targets of the kind listed here.

More recently, pangenome analysis across the *K. pneumoniae* species demonstrated substantial metabolic diversity associated with accessory gene content and lineage-specific adaptations. The first model-driven pangenome was published in 2019 (92), and 5 years later, the development of the KpSC pan-metabolic model derived from more than 500 genomes further extended this approach by incorporating accessory metabolic functions into a unified framework capable of generating high-quality strain-specific reconstructions (118).

A recent integrative study of comparative genomics, genome-scale metabolic modeling, machine learning, and metabolism and expression modeling investigated strain-specific metabolic diversity across the *Pseudomonas* genus. By reconstructing and analyzing 44 strain-specific GEMs and simulating growth under 425 nutrient conditions, the authors identified metabolic capabilities that differentiate species and provided mechanistic insights into proteome allocation and cell envelope-associated pathways. Overall, the study provides metabolic markers that are consistent for different *Pseudomonas aeruginosa* strains, such as glycerol-3-phosphate, L-leucine, and shikimate, among others (24).

Sequence-driven pangenome analyses of hundreds of genomes have also been performed, revealing new accessory genes, mobile elements, and novel loci (119, 120), as well as aiding in the definition of clones (e.g., *P. aeruginosa* ST235/ST111/ST175, *A. baumannii* Global Clones 1 and 2, and *K. pneumoniae* KpSC [species complex] with hypervirulent ST23 and MDR lineages) (40, 121–126). It has been observed that plasmids can carry nearly five times as many AMR genes as chromosomes, whereas virulence genes are more likely to be found on chromosomal “masked” genomes (12). The roles of many accessory and unique genes identified in these pangenome studies remain unknown or poorly characterized (115).

Overall, pangenomes serve as maps of genetic potential, while multi-strain genome-scale models convert that potential into predicted phenotypes. As sequencing and modeling tools continue to advance, these approaches will only grow in power and relevance, especially given the persistent threat of antibiotic resistance documented across the ESKAPEE group (51).

## MACHINE LEARNING CAN RAPIDLY IDENTIFY AND RANK KEY GENETIC TRAITS

The rapid growth of genomic sequencing has generated massive data sets for studying pathogens, but extracting meaningful biological insights from such data require powerful computational approaches. Machine learning (ML) has emerged as a critical tool in this effort, enabling researchers to model complex relationships between genotypes, phenotypes, and host interactions. Recent studies illustrate how ML can advance our understanding of pathogen biology, from predicting antimicrobial resistance to reconstructing regulatory networks and exploring population-level diversity.

One of the most direct applications of ML is predicting antimicrobial resistance from genome sequences by integrating thousands of features simultaneously, enabling the identification of subtle patterns in large-scale genomic data sets. Two distinct prediction tasks are usually conflated here. Genotypic prediction asks whether a genome carries known or inferred resistance determinants and whether classifiers trained on gene presence, single-nucleotide variants, or k-mer content perform this well across species (127, 128). Phenotypic prediction is harder since it requires estimating a susceptibility category or a minimum inhibitory concentration from sequence alone. Models of this second type have been built for several ESKAPE pathogens: for *K. pneumoniae*, an *in silico* MIC panel was constructed across multiple antibiotic classes (129); for *P. aeruginosa*, classifiers combining genomic and transcriptomic features predicted resistance for clinical diagnostic use (130); and comparable approaches have been reported for *S. aureus* (131) and *A. baumannii* (132). Performance is consistently strongest for antibiotics whose resistance is dominated by a small number of acquired determinants and weakest where resistance is polygenic or regulatory, which is precisely where model-derived flux features may add information the genome sequence alone does not carry.

Beyond resistance, ML can be used to uncover the regulatory processes that pathogens employ to adapt to all experimentally characterized environments to date. By leveraging transcriptomic data, ML algorithms have been applied to prototype gene regulatory networks, providing insight into how bacteria rewire their biology using unsupervised learning. These analyses are available in iModulonDB for *S. aureus*, *A. baumannii*, and *P. aeruginosa*. Genes are organized into iModulons, which are proxies for clusters of genes regulated by a transcription factor (133). They simplify understanding and visualization of complex regulatory processes, such as those mediated by virulence factors in *S. aureus* or multidrug efflux systems in *P. aeruginosa*. iModulons are generated from all available high-quality expression data often collected over decades of experimental work. Currently, further understanding of new gene functions using this method is limited by data availability. However, metabolic modeling can help expand the source data needed for AI/ML applications. Such methods not only improve understanding of basic biology but also identify potential vulnerabilities that could be targeted in therapeutic design.

While challenges remain, particularly regarding model interpretability and the need for high-quality training data sets, the trajectory of recent work is clear. ML is reshaping how we study and combat pathogens, and metabolic modeling can further empower it to pass from descriptive microbiology into predictive and intervention-oriented science.

## HOST AND POLYMICROBIAL INTERACTIONS AS ADVANCED MODELING APPLICATIONS

Host association and polymicrobial context are the most demanding applications of the modeling framework described above because they require representing metabolism in more than one organism at once and accounting for the exchange of metabolites between them. ESKAPE pathogens frequently originate as commensals or from the hospital environment before breaching physical barriers (134, 135), and their interactions span competition through mutualism, most often within multi-species biofilms at mucosal or wound sites (136, 137). A range of experimental systems has been developed to characterize host responses, from animal infection models to *in vitro* and *ex vivo* human systems (138, 139). Tables S1 and S2 summarize the most commonly used models together with the genome-scale reconstructions and omics data currently available for each host. These are illustrative rather than exhaustive: the published literature on host phenotypes in response to ESKAPE infection is far larger than any table can represent, and the entries were selected to indicate the range of systems in use and the modeling resources that exist for them.

What makes these settings tractable for metabolic modeling is that much of the relevant adaptation is metabolic. ESKAPE species persist in host niches by adjusting metabolism to acidic pH, nutrient limitation, and oxidative stress: *P. aeruginosa* exploits the succinate-rich environment of the inflamed lung; *S. aureus* shifts to alternative carbon sources under nutrient deprivation; and *A. baumannii* tolerates desiccation across a range of conditions (140). Some cells enter a dormant persister state that survives both antibiotics and immune clearance and underlies recurrent infection (56, 141). These are precisely the shifts a condition-specific model is built to predict, provided the corresponding expression or metabolomic data exist.

Extending models to polymicrobial communities is harder, and the principal difficulty is representing cell-to-cell metabolite exchange rather than any single organism’s metabolism (142). Progress has come mostly from paired and small-community systems. Communication between *S. aureus* and *S. epidermidis* was simulated using a domain-decomposition approach, yielding a Nash equilibrium model of biofilm formation involving 33 exchanged metabolites and accounting for their spatial distribution (143). Cystic fibrosis co-culture systems have received the most attention: community models predicted that interactions shift depending on whether *Staphylococcus* and *Pseudomonas* are present, with mutualistic pairings among *Staphylococcus*/*Acinetobacter*, *Pseudomonas*/*Serratia*, and *Streptococcus*/*Enterococcus* predicted in their absence (144), consistent with the observed strain dependence of *P. aeruginosa* lethality toward *S. aureus* (145). Related work predicted amensalistic interactions between *K. pneumoniae* and *Aspergillus* and *Penicillium* species (146), and *E. faecalis* biofilms have been shown to influence *Candida albicans* during co-colonization (147). On the host side, the only validated genome-scale host-pathogen model to date is for *M. tuberculosis* (148), which indicates how far this application remains from routine use for ESKAPE organisms. No single system recapitulates all aspects of ESKAPE infection, and multiple platforms are generally combined (138, 149).

## CONCLUSIONS AND FUTURE PERSPECTIVES

The scope of this review is deliberately restricted to the ESKAPE group, but the modeling framework described here is not. The WHO priority list extends well beyond ESKAPE, and the organisms named on it represent only a fraction of the clinically relevant resistant taxa. Genome-scale reconstructions already exist for several of them, including *Mycobacterium tuberculosis* and *Rothia mucilaginosa* (148, 150), and the same reconstruction, contextualization, and machine-learning steps described above apply directly to any organism with a sequenced genome. This is what makes the approach scalable as new priority pathogens emerge. Complementary formalisms are also being developed for questions that flux-based models handle poorly. Membrane computing, for instance, represents resistance evolution across nested ecological compartments (cell, patient, ward, hospital, community) and can simulate the transmission and selection dynamics that a single-organism metabolic model cannot capture (151, 152). Coupling compartmental frameworks of this kind to genome-scale reconstructions of the organisms within them is an open and largely unexplored direction. Realizing this will depend on automated generation of high-quality reconstructions since model availability, not modeling theory, is the current bottleneck.

## SUPPLEMENTAL MATERIAL

Supplemental material iai.00435-26-s0001.docx Tables S1 and S2.

ASM does not own the copyrights to Supplemental Material that may be linked to, or accessed through, an article. The authors have granted ASM a non-exclusive, world-wide license to publish the Supplemental Material files. Please contact the corresponding author directly for reuse.
