# Supplementary material for: Metabolic modeling: the link between AI and multi-omics data to accelerate the discovery of antibiotic-resistant traits in ESKAPE pathogens
Source: Infect Immun. 2026 Oct 7;94(11):e00435-26. doi: 10.1128/iai.00435-26 (PMC13750124; doi:10.1128/iai.00435-26)
Supplement: Supplementary file 1 [file iai.00435-26-s0001.docx]

**SUPPLEMENTAL TABLE S1** Experimental mammalian models used to study ESKAPE pathogens.

| **Model organism** | **Typical ESKAPE use** | **Multi-omics data** | **Key strengths** | **Main limitations** | **References** |
| --- | --- | --- | --- | --- | --- |
| **Mouse (*Mus musculus*)**  BiGG ID:  iMM1415  (Reactions: 3726, Metabolites: 2775, Genes: 1375) (126) | *K. pneumoniae, P. aeruginosa, A. baumannii, S. aureus, E. faecium, Enterobacter spp.* | Available genomics, single-cell transcriptomics, epigenomics and proteomics in public archives. | Extensive immunological and genetic toolbox, many knockout/knock-in and humanized strains, standardized protocols | Murine immune and physiological differences from humans, some syndromes and drug responses do not fully recapitulate clinical disease. | (1–7) |
| **Rat (*Rattus norvegicus*)**  GEM_ID: Rat1  (Reactions: 13101, Metabolites: 8408, Genes: 3499) (134) | *K. pneumoniae*, *P. aeruginosa, S. aureus, A. baumannii* | Whole genome, RNA-seq, microarray, proteomic and metabolomic datasets | Well-established infection models, Larger size allows richer sampling. | Fewer genetic tools than mice, notable immune differences from humans remain. | (8–13) |
| **Guinea pig (*Cavia porcellus*)**  No BiGG model available | *P. aeruginosa* | Immunoglobulin genomics, a smaller set of transcriptome and proteome studies | In certain contexts, tissue and immune responses can more closely resemble humans. | Limited reagents and genomics data, higher cost and housing demands than mice, less standardized. | (14–16) |
| **Rabbit**  No BiGG model available | *S. aureus*, *E. faecium* | Reference genome exist and there are proteomics, transcriptomics datasets | Strong for pathophysiology and antibiotic Pharmacokinetics (PK)/ Pharmacodynamics (PD). | Higher cost and ethical constraints, limited genetic modification, more complex procedures. | (17–20) |
| **Pig (*Sus scrofa*)**  No BiGG model available | *S. aureus, P. aeruginosa* | High-quality reference genome and transcriptomes, many proteomics tissue atlases | Skin and physiology closely related to humans, highly translational for skin and soft-tissue infection and device infection. | Very expensive, demanding housing and surgical/anesthesia support, low throughput. | (14, 21–25) |

**SUPPLEMENTAL TABLE S2** Other non-mammalian models used for ESKAPE pathogens

| Model organism | ESKAPE examples | **Multi-omics data** | Key strengths | Main limitations | References |
| --- | --- | --- | --- | --- | --- |
| *Caenorhabditis elegans*  GEM_ID: Worm1  (Reactions: 12201, Metabolites: 8183, Genes: 1949) (134) | *P. aeruginosa*, *Klebsiella* spp., *Enterobacter* spp. | High-quality reference genome, transcriptomics and proteomics data | High-throughput, inexpensive, strong genetics, conserved innate immunity. | No adaptive immunity, invertebrate anatomy, poor PK/PD translation. | (26–30) |
| *Galleria mellonella* (Wax moth larvae) | *A. baumannii*, *P. aeruginosa*, *K. pneumoniae*, *S. aureus*, *E. faecium* | Available draft genome, transcriptomics data | Incubation at 37 °C, easy handling, rapid virulence and efficacy screening with low ethical burden. | No adaptive immunity, limited genetic tools, partially defined immunity, batch variability in larvae. | (31–34) |
| *Drosophila melanogaster*  GEM_ID: Fruitfly1  (Reactions: 12072, Metabolites: 8164, Genes: 2048) (134) | *S. aureus*, *E. faecium*, *P. aeruginosa*. | Gold-standard reference genome, transcriptomics and proteomics data | Powerful genetic tools, suited to host-side mechanistic work. | Lacks adaptive immunity, physiology distant from humans, dosing less standardized. | (35–38) |
| *Bombyx mori* (silkworm) | Drug-resistant clinical isolates, including ESKAPE-like strains. | Available draft genome, transcriptomics data | Cheap, scalable, useful for early in vivo assessment of high-risk pathogens and compounds. | Invertebrate immunity, few reagents, mainly qualitative clinical extrapolation. | (2, 39–41) |
| Zebrafish (*Danio rerio*)  GEM_ID: Zebrafish1  (Reactions: 12956, Metabolites: 8384, Genes: 3228) (134) | *P. aeruginosa*, *A. baumannii*, *Enterobacter* spp. | High-quality genome, transcriptomics and Proteomics data | Vertebrate innate/adaptive immunity, optical transparency, genetic tractability. | Lower temperature and distinct physiology, PK/PD and pathology require careful translation. | (42–45) |
| *Ex vivo* human models (human cell line, organoids)  BIGG ID: RECON1  (Reactions: 10600, Metabolites: 5835, Genes: 2248) (126) | All ESKAPE pathogens | High-quality reference genome, transcriptomics and proteomics data | Best used for adhesion, invasion, toxin activity, biofilms, early immune signaling | Not sufficient for sepsis, chronic infection, resistance evolution, transmission | (34, 46–50) |

Information for Timeline Figure 2.

*Enterococcus faecium*

| Year | Events | Reference |
| --- | --- | --- |
| 1986–1988 | First outbreaks caused by vancomycin-resistant E. faecium (VREfm) reported in the United Kingdom | (51) |
| 1988 | Plasmids identified as carriers of vancomycin resistance (vanA type) | (52) |
| 2002 | Multilocus sequence typing (MLST) scheme introduced, enabling precise tracking of global genetic lineages. | (53) |
| 2005 | Global spread of clonal complex CC17 | (54) |
| 2012 | First genome sequence for *E. faecium* DO (TX0016) | (55) |
| 2012 | E. faecium population structure divided into two major clades (A and B) with A1 linked to clinical isolates | (56, 57) |
| 2015 | Introduction of core genome MLST (cgMLST) and multi-clade population structure confirmed | (58) |
| 2017 | WHO declares High Priority pathogen (vancomycin-resistant) | (59) |
| 2026 | First GEM | (60) |

*Staphylococcus aureus*

| Year | Events | Reference |
| --- | --- | --- |
| 1961 | First Methicillin-resistant S. aureus (MRSA) reported in the UK | (61) |
| 1968 | MRSA reported in hospitals in the USA | (62) |
| 1980 | MRSA becomes endemic in hospitals worldwide | (63) |
| 2001 | First complete genome sequence of MRSA strain N315 | (64) |
| 2005 | First GEM | (65) |
| 2007 | USA CDC estimates 94,000 severe MRSA infections/year | (66) |
| 2003–2008 | Emergence of livestock-associated MRSA (ST398) in Europe | (67) |
| 2015 | Complete genome sequencing of S. aureus type strain Rosenbach 1884 (DSM 20231T) published, setting a standard reference for population genomics. | (68) |
| 2016 | First model-driven pangenome analysis of 64 strains | (69) |
| 2021 | Curation and comparison of 114 published strain-specific GEMs | (70) |

*Klebsiella pneumoniae*

| Year | Events | Reference |
| --- | --- | --- |
| 1980 | *K. pneumoniae* becomes a leading producer of TEM- and SHV-derived ESBLs in hospitals worldwide, driving third-generation cephalosporin resistance and outbreaks. | (71) |
| 1996 | The first KPC-producing isolate identified was a ST37 strain collected in 1996 in North Carolina, USA | (72) |
| 2000-2003 | KPC-producing ST258 likely originated in the northeastern United States | (73) |
| 2007 | First complete genome sequence of MGH 78578 | (74) |
| 2007 | First reported outbreak of OXA-48-producing carbapenem-resistant *K. pneumoniae*, at a university hospital in Istanbul. | (75) |
| 2008 | The first NDM-1 enzyme was identified in 2008 in an extensively drug-resistant K. pneumoniae isolate from the urine of a Swedish patient of Indian origin who had received medical care in New Delhi; this isolate was described as sequence type ST14. | (76) |
| 2011 | First GEM | (77) |
| 2015 | From China, mcr-1 was identified on a transferable plasmid in *Escherichia coli* from pigs, raw meat, and humans | (78) |
| 2018–2019 | Emergence and rapid global dissemination of CTX-M-15-associated *Klebsiella pneumoniae* ST307” formally defines ST307 as a rapidly spreading, globally disseminated MDR clone | (79) |
| 2019 | First model-driven pangenome analysis of 22 strains | (80) |
| 2024 | Second model-driven pangenome analysis 507 strains | (81) |

*Acinetobacter baumannii*

| Year | Events | Reference |
| --- | --- | --- |
| After about 1975-1980 | Resistance to ampicillin, carbenicillin, gentamicin, and nalidixic acid was first reported (MDR) | (82) |
| 1985 | The first carbapenem-resistant *A. baumannii* (CRAB) was documented in 1985 in a patient at Edinburgh Royal Infirmary, essentially coinciding with imipenem’s clinical introduction | (83) |
| 1986 | The specific species name *A. baumannii* was then formally designated based on DNA–DNA hybridization work by Bouvet and Grimont | (84) |
| mid‑1990s | The first *Acinetobacter baumannii* “European clones” I–III (now called international clones IC1–IC3 or global clones GC1–GC3) | (85) |
| 2000s–2010s | International clone II (often ST2 by MLST) emerged as one of the most successful global CRAB lineages, frequently carrying blaOXA‑23 on transposons such as Tn2006 and driving numerous hospital outbreaks. | (86) |
| 2008 | First complete sequence of AYE | (87) |
| 2010 | First GEM | (88) |
| 2010 | NDM-1 in Acinetobacter baumannii was first reported | (89) |
| 2017 | WHO declares Critical Priority pathogen | (59) |

*Pseudomonas aeruginosa*

| Year | Events | Reference |
| --- | --- | --- |
| 1960s | Recognized as a major cause of hospital-acquired infections, particularly in burns, ICUs, and ventilator-associated pneumonia, notable for intrinsic resistance via low outer-membrane permeability, inducible AmpC, and efflux pumps. | (90) |
| 1990s | Carbapenem-resistant *P. aeruginosa* was increasingly reported in hospitals | (91) |
| In the 1990s and early 2000s | Acquired metallo-β-lactamases (MBLs) such as IMP- and VIM-type enzymes were identified in P. aeruginosa, conferring high-level carbapenem resistance and driving difficult-to-control outbreaks in many regions | (92) |
| 2000 | First complete genome sequence of PAO1 | (93) |
| 2008 | First GEM | (94) |
| 2011 | The first report of an NDM-1–producing *P. aeruginosa* is generally credited to a clinical isolate described from Serbia in 2011 | (95) |
| 2012 | First reported XDR *P. aeruginosa* co-producing VIM-2, GES-1, and OXA-1 β-lactamases | (96) |
| 2017 | *Pseudomonas aeruginosa* was first classified as a WHO priority pathogen | (59) |
| 2026 | First model-driven pangenome analysis 11 strains, and metabolism-and expression models | (97) |

*Enterobacter spp.*

| Year | Events | Reference |
| --- | --- | --- |
| 1960 | *Enterobacter* genus formally described by Hormaeche and Edwards, encompassing motile Gram-negative rods like *E. cloacae* (hospital isolates) | (98) |
| 1970 | Frequent outbreaks in ICUs; *E. cloacae* noted for "derepressed" AmpC mutants stable under cephalosporin pressure. | (99) |
| 1990s | CTX-M, SHV ESBLs reported; chromosomal AmpC + porin loss yields carbapenem resistance in some strains | (100, 101) |
| 1990s | *Enterobacter* becomes third most common nosocomial Gram-negative after *Pseudomonas* and *E. coli*; cefotaxime/ceftazidime introduced but AmpC limits efficacy. | (102) |
| 1991 | First ESBL-producing *Enterobacter* (*E. cloacae* with TEM-derived enzyme) | (103) |
| Mid-2000s | KPC-producing *E. cloacae* reported; plasmid-mediated carbapenemases spread. | (104) |
| 2010 | First complete genome sequence of *Enterobacter cloacae* subsp. *cloacae* type strain ATCC 13047 | (105) |
| 2015–2016 | *E. xiangfangensis* ST171 emerges as global carbapenem-resistant clone (mostly KPC). | (106) |
| 2022 | *CRE Enterobacter cases spike worldwide* | (107) |

**References**

1.	Chinwalla AT, Cook LL, Delehaunty KD, Fewell GA, Fulton LA, Fulton RS, Graves TA, Hillier LW, Mardis ER, McPherson JD, Miner TL, Nash WE, Nelson JO, Nhan MN, Pepin KH, Pohl CS, Ponce TC, Schultz B, Thompson J, Trevaskis E, Waterston RH, Wendl MC, Wilson RK, Yang S-P, An P, Berry E, Birren B, Bloom T, Brown DG, Butler J, Daly M, David R, Deri J, Dodge S, Foley K, Gage D, Gnerre S, Holzer T, Jaffe DB, Kamal M, Karlsson EK, Kells C, Kirby A, Kulbokas EJ, Lander ES, Landers T, Leger JP, Levine R, Lindblad-Toh K, Mauceli E, Mayer JH, McCarthy M, Meldrim J, Meldrim J, Mesirov JP, Nicol R, Nusbaum C, Seaman S, Sharpe T, Sheridan A, Singer JB, Santos R, Spencer B, Stange-Thomann N, Vinson JP, Wade CM, Wierzbowski J, Wyman D, Zody MC, Birney E, Goldman N, Kasprzyk A, Mongin E, Rust AG, Slater G, Stabenau A, Ureta-Vidal A, Whelan S, Ainscough R, Attwood J, Bailey J, Barlow K, Beck S, Burton J, Clamp M, Clee C, Coulson A, Cuff J, Curwen V, Cutts T, Davies J, Eyras E, Grafham D, Gregory S, Hubbard T, Hunt A, Jones M, Joy A, Leonard S, Lloyd C, Matthews L, McLaren S, McLay K, Meredith B, Mullikin JC, Ning Z, Oliver K, Overton-Larty E, Plumb R, Potter S, Quail M, Rogers J, Scott C, Searle S, Shownkeen R, Sims S, Wall M, West AP, Willey D, Williams S, Abril JF, Guigó R, Parra G, Agarwal P, Agarwala R, Church DM, Hlavina W, Maglott DR, Sapojnikov V, Alexandersson M, Pachter L, Antonarakis SE, Dermitzakis ET, Reymond A, Ucla C, Baertsch R, Diekhans M, Furey TS, Hinrichs A, Hsu F, Karolchik D, Kent WJ, Roskin KM, Schwartz MS, Sugnet C, Weber RJ, Bork P, Letunic I, Suyama M, Torrents D, Zdobnov EM, Botcherby M, Brown SD, Campbell RD, Jackson I, Bray N, Couronne O, Dubchak I, Poliakov A, Rubin EM, Brent MR, Flicek P, Keibler E, Korf I, Batalov S, Bult C, Frankel WN, Carninci P, Hayashizaki Y, Kawai J, Okazaki Y, Cawley S, Kulp D, Wheeler R, Chiaromonte F, Collins FS, Felsenfeld A, Guyer M, Peterson J, Wetterstrand K, Copley RR, Mott R, Dewey C, Dickens NJ, Emes RD, Goodstadt L, Ponting CP, Winter E, Dunn DM, von Niederhausern AC, Weiss RB, Eddy SR, Johnson LS, Jones TA, Elnitski L, Kolbe DL, Eswara P, Miller W, O’Connor MJ, Schwartz S, Gibbs RA, Muzny DM, Glusman G, Smit A, Green ED, Hardison RC, Yang S, Haussler D, Hua A, Roe BA, Kucherlapati RS, Montgomery KT, Li J, Li M, Lucas S, Ma B, McCombie WR, Morgan M, Pevzner P, Tesler G, Schultz J, Smith DR, Tromp J, Worley KC, Lander ES, Abril JF, Agarwal P, Alexandersson M, Antonarakis SE, Baertsch R, Berry E, Birney E, Bork P, Bray N, Brent MR, Brown DG, Butler J, Bult C, Chiaromonte F, Chinwalla AT, Church DM, Clamp M, Collins FS, Copley RR, Couronne O, Cawley S, Cuff J, Curwen V, Cutts T, Daly M, Dermitzakis ET, Dewey C, Mouse Genome Sequencing Consortium, Genome Sequencing Center:, Whitehead Institute/MIT Center for Genome Research:, European Bioinformatics Institute:, Wellcome Trust Sanger Institute, Research Group in Biomedical Informatics, Bioinformatics, National Center for Biotechnology Information, Department of Mathematics, Division of Medical Genetics, Center for Biomolecular Science and Engineering, EMBL, UK MRC Mouse Sequencing Consortium, Lawrence Berkeley National Laboratory, Department of Computer Science, School of Computer Science, The Jackson Laboratory, Laboratory for Genome Exploration, Affymetrix Inc., Departments of Statistics and Health Evaluation Sciences, National Human Genome Research Institute, Wellcome Trust Centre for Human Genetics, Department of Electrical Engineering, Department of Human Anatomy and Genetics, Department of Human Genetics, Howard Hughes Medical Institute and Department of Genetics, Departments of Biochemistry and Molecular Biology and Computer Science and Engineering, Department of Computer Science and Engineering, Baylor College of Medicine, The Institute for Systems Biology, Department of Biochemistry and Molecular Biology, Howard Hughes Medical Institute, Department of Chemistry and Biochemistry, Departments of Genetics and Medicine and Harvard-Partners Center for Genetics and Genomics, Department of Statistics, US DOE Joint Genome Institute, Cold Spring Harbor Laboratory, Wellcome Trust, Max Planck Institute for Molecular Genetics, Genome Therapeutics Corporation, Bioinformatics Solutions Inc., Department of Molecular and Human Genetics, Department of Biology, Members of the Mouse Genome Analysis Group. 2002. Initial sequencing and comparative analysis of the mouse genome. Nature 420:520–562.

2.	Yu H, Xu Y, Imani S, Zhao Z, Ullah S, Wang Q. 2024. Navigating ESKAPE Pathogens: Considerations and Caveats for Animal Infection Models Development. ACS Infect Dis 10:2336–2355.

3.	Paczosa MK, Mecsas J. 2016. Klebsiella pneumoniae: Going on the Offense with a Strong Defense. Microbiol Mol Biol Rev 80:629–661.

4.	Lyczak JB, Cannon CL, Pier GB. 2002. Lung Infections Associated with Cystic Fibrosis. Clin Microbiol Rev 15:194–222.

5.	Arias CA, Murray BE. 2012. The rise of the Enterococcus: beyond vancomycin resistance. Nat Rev Microbiol 10:266–278.

6.	Mortazavi A, Williams BA, McCue K, Schaeffer L, Wold B. 2008. Mapping and quantifying mammalian transcriptomes by RNA-Seq. Nat Methods 5:621–628.

7.	Geiger T, Velic A, Macek B, Lundberg E, Kampf C, Nagaraj N, Uhlen M, Cox J, Mann M. 2013. Initial quantitative proteomic map of 28 mouse tissues using the SILAC mouse. Mol Cell Proteomics 12:1709–1722.

8.	Hickman DL, Johnson J, Vemulapalli TH, Crisler JR, Shepherd R. 2017. Commonly Used Animal Models. Principles of Animal Research for Graduate and Undergraduate Students 117–175.

9.	Bengoechea JA, Sa Pessoa J. 2018. Klebsiella pneumoniae infection biology: living to counteract host defences. FEMS Microbiol Rev 43:123–144.

10.	Menzies BE. 2003. The role of fibronectin binding proteins in the pathogenesis of Staphylococcus aureus infections. Current Opinion in Infectious Diseases 16:225.

11.	Gibbs RA, Weinstock GM, Metzker ML, Muzny DM, Sodergren EJ, Scherer S, Scott G, Steffen D, Worley KC, Burch PE, Okwuonu G, Hines S, Lewis L, DeRamo C, Delgado O, Dugan-Rocha S, Miner G, Morgan M, Hawes A, Gill R, Holt CRA, Adams MD, Amanatides PG, Baden-Tillson H, Barnstead M, Chin S, Evans CA, Ferriera S, Fosler C, Glodek A, Gu Z, Jennings D, Kraft CL, Nguyen T, Pfannkoch CM, Sitter C, Sutton GG, Venter JC, Woodage T, Smith D, Lee H-M, Gustafson E, Cahill P, Kana A, Doucette-Stamm L, Weinstock K, Fechtel K, Weiss RB, Dunn DM, Green ED, Blakesley RW, Bouffard GG, de Jong PJ, Osoegawa K, Zhu B, Marra M, Schein J, Bosdet I, Fjell C, Jones S, Krzywinski M, Mathewson C, Siddiqui A, Wye N, McPherson J, Zhao S, Fraser CM, Shetty J, Shatsman S, Geer K, Chen Y, Abramzon S, Nierman WC, Gibbs RA, Weinstock GM, Havlak PH, Chen R, James Durbin K, Simons R, Ren Y, Song X-Z, Li B, Liu Y, Qin X, Cawley S, Weinstock (Coordinator) GM, Worley (Overall Coordinator) KC, Cooney AJ, Gibbs RA, D’Souza LM, Martin K, Qian Wu J, Gonzalez-Garay ML, Jackson AR, Kalafus KJ, McLeod MP, Milosavljevic A, Virk D, Volkov A, Wheeler DA, Zhang Z, Bailey JA, Eichler EE, Tuzun E, Birney E, Mongin E, Ureta-Vidal A, Woodwark C, Zdobnov E, Bork P, Suyama M, Torrents D, Alexandersson M, Trask BJ, Young JM, Smith D, Huang H, Fechtel K, Wang H, Xing H, Weinstock K, Daniels S, Gietzen D, Schmidt J, Stevens K, Vitt U, Wingrove J, Camara F, Mar Albà M, Abril JF, Guigo R, Smit A, Dubchak I, Rubin EM, Couronne O, Poliakov A, Hübner N, Ganten D, Goesele C, Hummel O, Kreitler T, Lee Y-A, Monti J, Schulz H, Zimdahl H, Himmelbauer H, Lehrach H, Jacob (Principal Investigator) HJ, Bromberg S, Gullings-Handley J, Jensen-Seaman MI, Kwitek AE, Lazar J, Pasko D, Tonellato PJ, Twigger S, Ponting CP, Duarte JM, Rice S, Goodstadt L, Beatson SA, Emes RD, Winter EE, Webber C, Brandt P, Nyakatura G, Adetobi M, Chiaromonte F, Elnitski L, Eswara P, Hardison RC, Hou M, Kolbe D, Makova K, Miller W, Nekrutenko A, Riemer C, Schwartz S, Taylor J, Yang S, Zhang Y, Lindpaintner K, Andrews TD, Caccamo M, Clamp M, Clarke L, Curwen V, Durbin R, Eyras E, Searle SM, Cooper GM, Batzoglou S, Brudno M, Sidow A, Stone EA, Craig Venter J, Payseur BA, Bourque G, López-Otín C, Puente XS, Chakrabarti K, Chatterji S, Dewey C, Pachter L, Bray N, Yap VB, Caspi A, Tesler G, Pevzner PA, Haussler D, Roskin KM, Baertsch R, Clawson H, Furey TS, Hinrichs AS, Karolchik D, Kent WJ, Rosenbloom KR, Trumbower H, Weirauch M, Cooper DN, Stenson PD, Ma B, Brent M, Arumugam M, Shteynberg D, Copley RR, Taylor MS, Riethman H, Mudunuri U, Peterson J, Guyer M, Felsenfeld A, Old S, Mockrin S, Collins F, Rat Genome Sequencing Project Consortium, DNA sequencing: Baylor College of Medicine, Genome Therapeutics, University of Utah, NISC Comparative Sequencing Program N, BAC library production: Children’s Hospital Oakland Research Institute, BAC fingerprinting: British Columbia Cancer Agency CMSGSC, Genome Sequencing Center WUS of M, BAC end sequencing: TIGR, Sequence assembly: Baylor College of Medicine, Analysis and annotation: Affymetrix, Baylor College of Medicine, Case Western Reserve University, EBI WTGC, EMBL H, Fraunhofer-Chalmers Research Centre for Industrial Mathematics G, Fred Hutchinson Cancer Research Center, Incyte Corporation, Institut Municipal d’Investigacio Medica B, The Institute for Systems Biology, Lawrence Berkeley National Laboratory, Max Delbrück Center for Molecular Medicine, Max Planck Institute for Molecular Genetics B, Medical College of Wisconsin, MRC Functional Genetics Unit U of O, MWG-Biotech, Pennsylvania State University, Roche Genetics and Roche Center for Medical Genomics, Sanger Institute, Stanford University, The Center for the Advancement of Genomics, University of Arizona, Université de Montréal, Universidad de Oviedo, University of California B, University of California SD, University of California SC, University of Wales College of Medicine, University of Western Ontario, Washington University, Wellcome Trust Centre for Human Genetics U of O, The Wistar Institute, Scientific management: 2004. Genome sequence of the Brown Norway rat yields insights into mammalian evolution. Nature 428:493–521.

12.	Yu Y, Fuscoe JC, Zhao C, Guo C, Jia M, Qing T, Bannon DI, Lancashire L, Bao W, Du T, Luo H, Su Z, Jones WD, Moland CL, Branham WS, Qian F, Ning B, Li Y, Hong H, Guo L, Mei N, Shi T, Wang KY, Wolfinger RD, Nikolsky Y, Walker SJ, Duerksen-Hughes P, Mason CE, Tong W, Thierry-Mieg J, Thierry-Mieg D, Shi L, Wang C. 2014. A rat RNA-Seq transcriptomic BodyMap across 11 organs and 4 developmental stages. Nat Commun 5:3230.

13.	Drastichova Z, Rudajev V, Pallag G, Novotny J. 2021. Proteome profiling of different rat brain regions reveals the modulatory effect of prolonged maternal separation on proteins involved in cell death-related processes. Biol Res 54:4.

14.	Sainz-Mejías M, Jurado-Martín I, McClean S. 2020. Understanding Pseudomonas aeruginosa–Host Interactions: The Ongoing Quest for an Efficacious Vaccine. Cells 9.

15.	Guo Y, Bao Y, Meng Q, Hu X, Meng Q, Ren L, Li N, Zhao Y. 2012. Immunoglobulin Genomics in the Guinea Pig (Cavia porcellus). PLoS One 7:e39298.

16.	Wali S, Gupta R, Veselenak RL, Li Y, Yu J-J, Murthy AK, Cap AP, Guentzel MN, Chambers JP, Zhong G, Rank RG, Pyles RB, Arulanandam BP. 2014. Use of a Guinea pig-specific transcriptome array for evaluation of protective immunity against genital chlamydial infection following intranasal vaccination in Guinea pigs. PLoS One 9:e114261.

17.	Baddour LM, Christensen GD, Hester MG, Bisno AL. 1984. Production of Experimental Endocarditis by Coagulase-Negative Staphylococci: Variability in Species Virulence. J Infect Dis 150:721–727.

18.	Bai Y, Lin W, Xu J, Song J, Yang D, Chen YE, Li L, Li Y, Wang Z, Zhang J. 2021. Improving the genome assembly of rabbits with long-read sequencing. Genomics 113:3216–3223.

19.	Wu J, Zhai J, Jia H, Ahamba IS, Dong X, Ren Z. 2025. Whole-transcriptome analysis reveals the profiles and roles of coding and non-coding RNAs during hair follicle cycling in Rex rabbits. BMC Genomics 26:74.

20.	Casares-Crespo L, Fernández-Serrano P, Viudes-de-Castro MP. 2019. Proteomic characterization of rabbit (Oryctolagus cuniculus) sperm from two different genotypes. Theriogenology 128:140–148.

21.	Nielsen OL, Mellergaard M, Frees D, Larsen MN, Skov S, Olsen LH, Reimann MJ. 2022. A porcine model of subcutaneous Staphylococcus aureus infection: a pilot study. APMIS 130:359–370.

22.	Davis SC, Li J, Gil J, Head C, Valdes J, Glinos GD, Solis M, Higa A, Pastar I. 2019. Preclinical evaluation of a novel silver gelling fiber dressing on Pseudomonas aeruginosa in a porcine wound infection model. Wound Repair Regen 27:360–365.

23.	Warr A, Affara N, Aken B, Beiki H, Bickhart DM, Billis K, Chow W, Eory L, Finlayson HA, Flicek P, Girón CG, Griffin DK, Hall R, Hannum G, Hourlier T, Howe K, Hume DA, Izuogu O, Kim K, Koren S, Liu H, Manchanda N, Martin FJ, Nonneman DJ, O’Connor RE, Phillippy AM, Rohrer GA, Rosen BD, Rund LA, Sargent CA, Schook LB, Schroeder SG, Schwartz AS, Skinner BM, Talbot R, Tseng E, Tuggle CK, Watson M, Smith TPL, Archibald AL. 2020. An improved pig reference genome sequence to enable pig genetics and genomics research. Gigascience 9:giaa051.

24.	Jin L, Tang Q, Hu S, Chen Z, Zhou X, Zeng B, Wang Y, He M, Li Y, Gui L, Shen L, Long K, Ma J, Wang X, Chen Z, Jiang Y, Tang G, Zhu L, Liu F, Zhang B, Huang Z, Li G, Li D, Gladyshev VN, Yin J, Gu Y, Li X, Li M. 2021. A pig BodyMap transcriptome reveals diverse tissue physiologies and evolutionary dynamics of transcription. Nat Commun 12:3715.

25.	Miller I, Wait R, Sipos W, Gemeiner M. 2009. A proteomic reference map for pig serum proteins as a prerequisite for diagnostic applications. Res Vet Sci 86:362–367.

26.	MARTINEAU CN, KIRIENKO NV, PUJOL N. 2021. INNATE IMMUNITY IN C. ELEGANS. Curr Top Dev Biol 144:309–351.

27.	Tan MW, Mahajan-Miklos S, Ausubel FM. 1999. Killing of Caenorhabditis elegans by Pseudomonas aeruginosa used to model mammalian bacterial pathogenesis. Proc Natl Acad Sci U S A 96:715–720.

28.	C. elegans Sequencing Consortium. 1998. Genome sequence of the nematode C. elegans: a platform for investigating biology. Science 282:2012–2018.

29.	Gerstein MB, Lu ZJ, Van Nostrand EL, Cheng C, Arshinoff BI, Liu T, Yip KY, Robilotto R, Rechtsteiner A, Ikegami K, Alves P, Chateigner A, Perry M, Morris M, Auerbach RK, Feng X, Leng J, Vielle A, Niu W, Rhrissorrakrai K, Agarwal A, Alexander RP, Barber G, Brdlik CM, Brennan J, Brouillet JJ, Carr A, Cheung M-S, Clawson H, Contrino S, Dannenberg LO, Dernburg AF, Desai A, Dick L, Dosé AC, Du J, Egelhofer T, Ercan S, Euskirchen G, Ewing B, Feingold EA, Gassmann R, Good PJ, Green P, Gullier F, Gutwein M, Guyer MS, Habegger L, Han T, Henikoff JG, Henz SR, Hinrichs A, Holster H, Hyman T, Iniguez AL, Janette J, Jensen M, Kato M, Kent WJ, Kephart E, Khivansara V, Khurana E, Kim JK, Kolasinska-Zwierz P, Lai EC, Latorre I, Leahey A, Lewis S, Lloyd P, Lochovsky L, Lowdon RF, Lubling Y, Lyne R, MacCoss M, Mackowiak SD, Mangone M, McKay S, Mecenas D, Merrihew G, Miller DM, Muroyama A, Murray JI, Ooi S-L, Pham H, Phippen T, Preston EA, Rajewsky N, Rätsch G, Rosenbaum H, Rozowsky J, Rutherford K, Ruzanov P, Sarov M, Sasidharan R, Sboner A, Scheid P, Segal E, Shin H, Shou C, Slack FJ, Slightam C, Smith R, Spencer WC, Stinson EO, Taing S, Takasaki T, Vafeados D, Voronina K, Wang G, Washington NL, Whittle CM, Wu B, Yan K-K, Zeller G, Zha Z, Zhong M, Zhou X, modENCODE Consortium, Ahringer J, Strome S, Gunsalus KC, Micklem G, Liu XS, Reinke V, Kim SK, Hillier LW, Henikoff S, Piano F, Snyder M, Stein L, Lieb JD, Waterston RH. 2010. Integrative analysis of the Caenorhabditis elegans genome by the modENCODE project. Science 330:1775–1787.

30.	Schrimpf SP, Weiss M, Reiter L, Ahrens CH, Jovanovic M, Malmström J, Brunner E, Mohanty S, Lercher MJ, Hunziker PE, Aebersold R, von Mering C, Hengartner MO. 2009. Comparative functional analysis of the Caenorhabditis elegans and Drosophila melanogaster proteomes. PLoS Biol 7:e48.

31.	Ménard G, Rouillon A, Cattoir V, Donnio P-Y. 2021. Galleria mellonella as a Suitable Model of Bacterial Infection: Past, Present and Future. Front Cell Infect Microbiol 11:782733.

32.	Lange A, Beier S, Huson DH, Parusel R, Iglauer F, Frick J-S. 2018. Genome Sequence of Galleria mellonella (Greater Wax Moth). Genome Announcements 6:10.1128/genomea.01220-17.

33.	Vogel H, Altincicek B, Glöckner G, Vilcinskas A. 2011. A comprehensive transcriptome and immune-gene repertoire of the lepidopteran model host Galleria mellonella. BMC Genomics 12:308.

34.	Muzahid NH, Ramesh A, Siew TH, Hasan MZ, Narayanan K, Rahman S. 2025. Comparison of the virulence of community- and hospital- isolated Acinetobacter baumannii in HeLa cell line and insect model, Galleria mellonella. Access Microbiol 7:000858.v3.

35.	Apidianakis Y, Rahme LG. 2011. Drosophila melanogaster as a model for human intestinal infection and pathology. Dis Model Mech 4:21–30.

36.	Adams MD, Celniker SE, Holt RA, Evans CA, Gocayne JD, Amanatides PG, Scherer SE, Li PW, Hoskins RA, Galle RF, George RA, Lewis SE, Richards S, Ashburner M, Henderson SN, Sutton GG, Wortman JR, Yandell MD, Zhang Q, Chen LX, Brandon RC, Rogers YH, Blazej RG, Champe M, Pfeiffer BD, Wan KH, Doyle C, Baxter EG, Helt G, Nelson CR, Gabor GL, Abril JF, Agbayani A, An HJ, Andrews-Pfannkoch C, Baldwin D, Ballew RM, Basu A, Baxendale J, Bayraktaroglu L, Beasley EM, Beeson KY, Benos PV, Berman BP, Bhandari D, Bolshakov S, Borkova D, Botchan MR, Bouck J, Brokstein P, Brottier P, Burtis KC, Busam DA, Butler H, Cadieu E, Center A, Chandra I, Cherry JM, Cawley S, Dahlke C, Davenport LB, Davies P, de Pablos B, Delcher A, Deng Z, Mays AD, Dew I, Dietz SM, Dodson K, Doup LE, Downes M, Dugan-Rocha S, Dunkov BC, Dunn P, Durbin KJ, Evangelista CC, Ferraz C, Ferriera S, Fleischmann W, Fosler C, Gabrielian AE, Garg NS, Gelbart WM, Glasser K, Glodek A, Gong F, Gorrell JH, Gu Z, Guan P, Harris M, Harris NL, Harvey D, Heiman TJ, Hernandez JR, Houck J, Hostin D, Houston KA, Howland TJ, Wei MH, Ibegwam C, Jalali M, Kalush F, Karpen GH, Ke Z, Kennison JA, Ketchum KA, Kimmel BE, Kodira CD, Kraft C, Kravitz S, Kulp D, Lai Z, Lasko P, Lei Y, Levitsky AA, Li J, Li Z, Liang Y, Lin X, Liu X, Mattei B, McIntosh TC, McLeod MP, McPherson D, Merkulov G, Milshina NV, Mobarry C, Morris J, Moshrefi A, Mount SM, Moy M, Murphy B, Murphy L, Muzny DM, Nelson DL, Nelson DR, Nelson KA, Nixon K, Nusskern DR, Pacleb JM, Palazzolo M, Pittman GS, Pan S, Pollard J, Puri V, Reese MG, Reinert K, Remington K, Saunders RD, Scheeler F, Shen H, Shue BC, Sidén-Kiamos I, Simpson M, Skupski MP, Smith T, Spier E, Spradling AC, Stapleton M, Strong R, Sun E, Svirskas R, Tector C, Turner R, Venter E, Wang AH, Wang X, Wang ZY, Wassarman DA, Weinstock GM, Weissenbach J, Williams SM, WoodageT null, Worley KC, Wu D, Yang S, Yao QA, Ye J, Yeh RF, Zaveri JS, Zhan M, Zhang G, Zhao Q, Zheng L, Zheng XH, Zhong FN, Zhong W, Zhou X, Zhu S, Zhu X, Smith HO, Gibbs RA, Myers EW, Rubin GM, Venter JC. 2000. The genome sequence of Drosophila melanogaster. Science 287:2185–2195.

37.	Graveley BR, Brooks AN, Carlson JW, Duff MO, Landolin JM, Yang L, Artieri CG, van Baren MJ, Boley N, Booth BW, Brown JB, Cherbas L, Davis CA, Dobin A, Li R, Lin W, Malone JH, Mattiuzzo NR, Miller D, Sturgill D, Tuch BB, Zaleski C, Zhang D, Blanchette M, Dudoit S, Eads B, Green RE, Hammonds A, Jiang L, Kapranov P, Langton L, Perrimon N, Sandler JE, Wan KH, Willingham A, Zhang Y, Zou Y, Andrews J, Bickel PJ, Brenner SE, Brent MR, Cherbas P, Gingeras TR, Hoskins RA, Kaufman TC, Oliver B, Celniker SE. 2011. The developmental transcriptome of Drosophila melanogaster. Nature 471:473–479.

38.	Brunner E, Ahrens CH, Mohanty S, Baetschmann H, Loevenich S, Potthast F, Deutsch EW, Panse C, de Lichtenberg U, Rinner O, Lee H, Pedrioli PGA, Malmstrom J, Koehler K, Schrimpf S, Krijgsveld J, Kregenow F, Heck AJR, Hafen E, Schlapbach R, Aebersold R. 2007. A high-quality catalog of the Drosophila melanogaster proteome. Nat Biotechnol 25:576–583.

39.	Kawamoto M, Jouraku A, Toyoda A, Yokoi K, Minakuchi Y, Katsuma S, Fujiyama A, Kiuchi T, Yamamoto K, Shimada T. 2019. High-quality genome assembly of the silkworm, Bombyx mori. Insect Biochem Mol Biol 107:53–62.

40.	Yang L, Gao Q, Dai J, Yuan G, Wang L, Qian C, Zhu B, Liu C, Wei G. 2018. Comparative transcriptome analysis of silkworm, Bombyx mori colleterial gland suggests their functional role in mucous secretion. PLOS ONE 13:e0198077.

41.	Zhang P, Aso Y, Yamamoto K, Banno Y, Wang Y, Tsuchida K, Kawaguchi Y, Fujii H. 2006. Proteome analysis of silk gland proteins from the silkworm, Bombyx mori. Proteomics 6:2586–2599.

42.	Torraca V, Mostowy S. 2018. Zebrafish Infection: From Pathogenesis to Cell Biology. Trends Cell Biol 28:143–156.

43.	Howe K, Clark MD, Torroja CF, Torrance J, Berthelot C, Muffato M, Collins JE, Humphray S, McLaren K, Matthews L, McLaren S, Sealy I, Caccamo M, Churcher C, Scott C, Barrett JC, Koch R, Rauch G-J, White S, Chow W, Kilian B, Quintais LT, Guerra-Assunção JA, Zhou Y, Gu Y, Yen J, Vogel J-H, Eyre T, Redmond S, Banerjee R, Chi J, Fu B, Langley E, Maguire SF, Laird GK, Lloyd D, Kenyon E, Donaldson S, Sehra H, Almeida-King J, Loveland J, Trevanion S, Jones M, Quail M, Willey D, Hunt A, Burton J, Sims S, McLay K, Plumb B, Davis J, Clee C, Oliver K, Clark R, Riddle C, Elliot D, Threadgold G, Harden G, Ware D, Begum S, Mortimore B, Kerry G, Heath P, Phillimore B, Tracey A, Corby N, Dunn M, Johnson C, Wood J, Clark S, Pelan S, Griffiths G, Smith M, Glithero R, Howden P, Barker N, Lloyd C, Stevens C, Harley J, Holt K, Panagiotidis G, Lovell J, Beasley H, Henderson C, Gordon D, Auger K, Wright D, Collins J, Raisen C, Dyer L, Leung K, Robertson L, Ambridge K, Leongamornlert D, McGuire S, Gilderthorp R, Griffiths C, Manthravadi D, Nichol S, Barker G, Whitehead S, Kay M, Brown J, Murnane C, Gray E, Humphries M, Sycamore N, Barker D, Saunders D, Wallis J, Babbage A, Hammond S, Mashreghi-Mohammadi M, Barr L, Martin S, Wray P, Ellington A, Matthews N, Ellwood M, Woodmansey R, Clark G, Cooper JD, Tromans A, Grafham D, Skuce C, Pandian R, Andrews R, Harrison E, Kimberley A, Garnett J, Fosker N, Hall R, Garner P, Kelly D, Bird C, Palmer S, Gehring I, Berger A, Dooley CM, Ersan-Ürün Z, Eser C, Geiger H, Geisler M, Karotki L, Kirn A, Konantz J, Konantz M, Oberländer M, Rudolph-Geiger S, Teucke M, Lanz C, Raddatz G, Osoegawa K, Zhu B, Rapp A, Widaa S, Langford C, Yang F, Schuster SC, Carter NP, Harrow J, Ning Z, Herrero J, Searle SMJ, Enright A, Geisler R, Plasterk RHA, Lee C, Westerfield M, de Jong PJ, Zon LI, Postlethwait JH, Nüsslein-Volhard C, Hubbard TJP, Roest Crollius H, Rogers J, Stemple DL. 2013. The zebrafish reference genome sequence and its relationship to the human genome. Nature 496:498–503.

44.	Pauli A, Valen E, Lin MF, Garber M, Vastenhouw NL, Levin JZ, Fan L, Sandelin A, Rinn JL, Regev A, Schier AF. 2012. Systematic identification of long noncoding RNAs expressed during zebrafish embryogenesis. Genome Res 22:577–591.

45.	Singh SK, Sundaram CS, Shanbhag S, Idris MM. 2010. Proteomic profile of zebrafish brain based on two-dimensional gel electrophoresis matrix-assisted laser desorption/ionization MS/MS analysis. Zebrafish 7:169–177.

46.	May S, Evans S, Parry L. 2017. Organoids, organs-on-chips and other systems, and microbiota. Emerg Top Life Sci 1:385–400.

47.	2026. Initial sequencing and analysis of the human genome | Nature. https://www.nature.com/articles/35057062. Retrieved 30 January 2026.

48.	Dunham I, Kundaje A, Aldred SF, Collins PJ, Davis CA, Doyle F, Epstein CB, Frietze S, Harrow J, Kaul R, Khatun J, Lajoie BR, Landt SG, Lee B-K, Pauli F, Rosenbloom KR, Sabo P, Safi A, Sanyal A, Shoresh N, Simon JM, Song L, Trinklein ND, Altshuler RC, Birney E, Brown JB, Cheng C, Djebali S, Dong X, Dunham I, Ernst J, Furey TS, Gerstein M, Giardine B, Greven M, Hardison RC, Harris RS, Herrero J, Hoffman MM, Iyer S, Kellis M, Khatun J, Kheradpour P, Kundaje A, Lassmann T, Li Q, Lin X, Marinov GK, Merkel A, Mortazavi A, Parker SCJ, Reddy TE, Rozowsky J, Schlesinger F, Thurman RE, Wang J, Ward LD, Whitfield TW, Wilder SP, Wu W, Xi HS, Yip KY, Zhuang J, Bernstein BE, Birney E, Dunham I, Green ED, Gunter C, Snyder M, Pazin MJ, Lowdon RF, Dillon LAL, Adams LB, Kelly CJ, Zhang J, Wexler JR, Green ED, Good PJ, Feingold EA, Bernstein BE, Birney E, Crawford GE, Dekker J, Elnitski L, Farnham PJ, Gerstein M, Giddings MC, Gingeras TR, Green ED, Guigó R, Hardison RC, Hubbard TJ, Kellis M, Kent WJ, Lieb JD, Margulies EH, Myers RM, Snyder M, Stamatoyannopoulos JA, Tenenbaum SA, Weng Z, White KP, Wold B, Khatun J, Yu Y, Wrobel J, Risk BA, Gunawardena HP, Kuiper HC, Maier CW, Xie L, Chen X, Giddings MC, Bernstein BE, Epstein CB, Shoresh N, Ernst J, Kheradpour P, Mikkelsen TS, Gillespie S, Goren A, Ram O, Zhang X, Wang L, Issner R, Coyne MJ, Durham T, Ku M, Truong T, Ward LD, Altshuler RC, Eaton ML, Kellis M, Djebali S, Davis CA, Merkel A, Dobin A, Lassmann T, Mortazavi A, Tanzer A, Lagarde J, Lin W, Schlesinger F, Xue C, Marinov GK, Khatun J, Williams BA, Zaleski C, Rozowsky J, Röder M, Kokocinski F, Abdelhamid RF, Alioto T, Antoshechkin I, Baer MT, Batut P, Bell I, Bell K, Chakrabortty S, Chen X, Chrast J, Curado J, Derrien T, Drenkow J, Dumais E, Dumais J, Duttagupta R, Fastuca M, Fejes-Toth K, Ferreira P, Foissac S, Fullwood MJ, Gao H, Gonzalez D, Gordon A, Gunawardena HP, Howald C, Jha S, Johnson R, Kapranov P, King B, Kingswood C, Li G, Luo OJ, Park E, Preall JB, Presaud K, Ribeca P, Risk BA, Robyr D, Ruan X, Sammeth M, Sandhu KS, Schaeffer L, See L-H, Shahab A, Skancke J, Suzuki AM, Takahashi H, Tilgner H, Trout D, Walters N, Wang H, Wrobel J, Yu Y, Hayashizaki Y, Harrow J, Gerstein M, Hubbard TJ, Reymond A, Antonarakis SE, Hannon GJ, Giddings MC, Ruan Y, Wold B, Carninci P, Guigó R, Gingeras TR, Rosenbloom KR, Sloan CA, Learned K, Malladi VS, Wong MC, Barber GP, Cline MS, Dreszer TR, Heitner SG, Karolchik D, Kent WJ, Kirkup VM, Meyer LR, Long JC, Maddren M, Raney BJ, Furey TS, Song L, Grasfeder LL, Giresi PG, Lee B-K, Battenhouse A, Sheffield NC, Simon JM, Showers KA, Safi A, London D, Bhinge AA, Shestak C, Schaner MR, Ki Kim S, Zhang ZZ, Mieczkowski PA, Mieczkowska JO, Liu Z, McDaniell RM, Ni Y, Rashid NU, Kim MJ, Adar S, Zhang Z, Wang T, Winter D, Keefe D, Birney E, Iyer VR, Lieb JD, Crawford GE, Li G, Sandhu KS, Zheng M, Wang P, Luo OJ, Shahab A, Fullwood MJ, Ruan X, Ruan Y, Myers RM, Pauli F, Williams BA, Gertz J, Marinov GK, Reddy TE, Vielmetter J, Partridge E, Trout D, Varley KE, Gasper C, The ENCODE Project Consortium, Overall coordination (data analysis coordination), Data production leads (data production), Lead analysts (data analysis), Writing group, NHGRI project management (scientific management), Principal investigators (steering committee), Boise State University and University of North Carolina at Chapel Hill Proteomics groups (data production and analysis), Broad Institute Group (data production and analysis), Cold Spring Harbor U of G Center for Genomic Regulation, Barcelona, RIKEN, Sanger Institute, University of Lausanne, Genome Institute of Singapore group (data production and analysis), Data coordination center at UC Santa Cruz (production data coordination), Duke University E University of Texas, Austin, University of North Carolina-Chapel Hill group (data production and analysis), Genome Institute of Singapore group (data production and analysis), HudsonAlpha Institute C UC Irvine, Stanford group (data production and analysis). 2012. An integrated encyclopedia of DNA elements in the human genome. Nature 489:57–74.

49.	GTEx Consortium. 2020. The GTEx Consortium atlas of genetic regulatory effects across human tissues. Science 369:1318–1330.

50.	Kim M-S, Pinto SM, Getnet D, Nirujogi RS, Manda SS, Chaerkady R, Madugundu AK, Kelkar DS, Isserlin R, Jain S, Thomas JK, Muthusamy B, Leal-Rojas P, Kumar P, Sahasrabuddhe NA, Balakrishnan L, Advani J, George B, Renuse S, Selvan LDN, Patil AH, Nanjappa V, Radhakrishnan A, Prasad S, Subbannayya T, Raju R, Kumar M, Sreenivasamurthy SK, Marimuthu A, Sathe GJ, Chavan S, Datta KK, Subbannayya Y, Sahu A, Yelamanchi SD, Jayaram S, Rajagopalan P, Sharma J, Murthy KR, Syed N, Goel R, Khan AA, Ahmad S, Dey G, Mudgal K, Chatterjee A, Huang T-C, Zhong J, Wu X, Shaw PG, Freed D, Zahari MS, Mukherjee KK, Shankar S, Mahadevan A, Lam H, Mitchell CJ, Shankar SK, Satishchandra P, Schroeder JT, Sirdeshmukh R, Maitra A, Leach SD, Drake CG, Halushka MK, Prasad TSK, Hruban RH, Kerr CL, Bader GD, Iacobuzio-Donahue CA, Gowda H, Pandey A. 2014. A draft map of the human proteome. Nature 509:575–581.

51.	Uttley AH, Collins CH, Naidoo J, George RC. 1988. Vancomycin-resistant enterococci. Lancet 1:57–58.

52.	Leclercq R, Derlot E, Duval J, Courvalin P. 1988. Plasmid-mediated resistance to vancomycin and teicoplanin in Enterococcus faecium. N Engl J Med 319:157–161.

53.	Homan WL, Tribe D, Poznanski S, Li M, Hogg G, Spalburg E, Van Embden JDA, Willems RJL. 2002. Multilocus sequence typing scheme for Enterococcus faecium. J Clin Microbiol 40:1963–1971.

54.	Willems RJL, Top J, van Santen M, Robinson DA, Coque TM, Baquero F, Grundmann H, Bonten MJM. 2005. Global Spread of Vancomycin-resistant Enterococcus faecium from Distinct Nosocomial Genetic Complex. Emerg Infect Dis 11:821–828.

55.	Qin X, Galloway-Peña JR, Sillanpaa J, Roh JH, Nallapareddy SR, Chowdhury S, Bourgogne A, Choudhury T, Muzny DM, Buhay CJ, Ding Y, Dugan-Rocha S, Liu W, Kovar C, Sodergren E, Highlander S, Petrosino JF, Worley KC, Gibbs RA, Weinstock GM, Murray BE. 2012. Complete genome sequence of Enterococcus faecium strain TX16 and comparative genomic analysis of Enterococcus faecium genomes. BMC Microbiol 12:135.

56.	Galloway-Peña J, Roh JH, Latorre M, Qin X, Murray BE. 2012. Genomic and SNP analyses demonstrate a distant separation of the hospital and community-associated clades of Enterococcus faecium. PLoS One 7:e30187.

57.	Palmer KL, Godfrey P, Griggs A, Kos VN, Zucker J, Desjardins C, Cerqueira G, Gevers D, Walker S, Wortman J, Feldgarden M, Haas B, Birren B, Gilmore MS. 2012. Comparative genomics of enterococci: variation in Enterococcus faecalis, clade structure in E. faecium, and defining characteristics of E. gallinarum and E. casseliflavus. mBio 3:e00318-00311.

58.	de Been M, Pinholt M, Top J, Bletz S, Mellmann A, van Schaik W, Brouwer E, Rogers M, Kraat Y, Bonten M, Corander J, Westh H, Harmsen D, Willems RJL. 2015. Core Genome Multilocus Sequence Typing Scheme for High- Resolution Typing of Enterococcus faecium. J Clin Microbiol 53:3788–3797.

59.	2024. WHO Bacterial Priority Pathogens List 2024: Bacterial Pathogens of Public Health Importance, to Guide Research, Development, and Strategies to Prevent and Control Antimicrobial Resistance1st ed. World Health Organization, Geneva.

60.	Rasmi DS, Krishnan J, Hashem YA, Palsson BO, Kashef MT, Monk JM, Aziz RK. 2026. The first digital twin of Enterococcus faecium metabolism reproduces high-throughput phenotyping data. bioRxiv https://doi.org/10.64898/2026.05.01.720924.

61.	Jevons MP. 1961. “Celbenin” - resistant Staphylococci. Br Med J 1:124–125.

62.	Barrett FF, McGehee RF, Finland M. 1968. Methicillin-resistant Staphylococcus aureus at Boston City Hospital. Bacteriologic and epidemiologic observations. N Engl J Med 279:441–448.

63.	Boyce JM, Causey WA. 1982. Increasing occurrence of methicillin-resistant Staphylococcus aureus in the United States. Infect Control 3:377–383.

64.	Kuroda M, Ohta T, Uchiyama I, Baba T, Yuzawa H, Kobayashi I, Cui L, Oguchi A, Aoki K, Nagai Y, Lian J, Ito T, Kanamori M, Matsumaru H, Maruyama A, Murakami H, Hosoyama A, Mizutani-Ui Y, Takahashi NK, Sawano T, Inoue R, Kaito C, Sekimizu K, Hirakawa H, Kuhara S, Goto S, Yabuzaki J, Kanehisa M, Yamashita A, Oshima K, Furuya K, Yoshino C, Shiba T, Hattori M, Ogasawara N, Hayashi H, Hiramatsu K. 2001. Whole genome sequencing of meticillin-resistant Staphylococcus aureus. Lancet 357:1225–1240.

65.	Becker SA, Palsson BØ. 2005. Genome-scale reconstruction of the metabolic network in Staphylococcus aureus N315: an initial draft to the two-dimensional annotation. BMC Microbiol 5:8.

66.	Klevens RM, Morrison MA, Nadle J, Petit S, Gershman K, Ray S, Harrison LH, Lynfield R, Dumyati G, Townes JM, Craig AS, Zell ER, Fosheim GE, McDougal LK, Carey RB, Fridkin SK, Active Bacterial Core surveillance (ABCs) MRSA Investigators. 2007. Invasive methicillin-resistant Staphylococcus aureus infections in the United States. JAMA 298:1763–1771.

67.	Smith TC, Pearson N. 2011. The emergence of Staphylococcus aureus ST398. Vector Borne Zoonotic Dis 11:327–339.

68.	Shiroma A, Terabayashi Y, Nakano K, Shimoji M, Tamotsu H, Ashimine N, Ohki S, Shinzato M, Teruya K, Satou K, Hirano T. 2015. First Complete Genome Sequences of Staphylococcus aureus subsp. aureus Rosenbach 1884 (DSM 20231T), Determined by PacBio Single-Molecule Real-Time Technology. Genome Announc 3:e00800-15.

69.	Bosi E, Monk JM, Aziz RK, Fondi M, Nizet V, Palsson BØ. 2016. Comparative genome-scale modelling of Staphylococcus aureus strains identifies strain-specific metabolic capabilities linked to pathogenicity. Proceedings of the National Academy of Sciences 113:E3801–E3809.

70.	Renz A, Dräger A. 2021. Curating and comparing 114 strain-specific genome-scale metabolic models of Staphylococcus aureus. npj Syst Biol Appl 7:30.

71.	Knothe H, Shah P, Krcmery V, Antal M, Mitsuhashi S. 1983. Transferable resistance to cefotaxime, cefoxitin, cefamandole and cefuroxime in clinical isolates of Klebsiella pneumoniae and Serratia marcescens. Infection 11:315–317.

72.	Yigit H, Queenan AM, Anderson GJ, Domenech-Sanchez A, Biddle JW, Steward CD, Alberti S, Bush K, Tenover FC. 2001. Novel carbapenem-hydrolyzing beta-lactamase, KPC-1, from a carbapenem-resistant strain of Klebsiella pneumoniae. Antimicrob Agents Chemother 45:1151–1161.

73.	Bowers JR, Kitchel B, Driebe EM, MacCannell DR, Roe C, Lemmer D, de Man T, Rasheed JK, Engelthaler DM, Keim P, Limbago BM. 2015. Genomic Analysis of the Emergence and Rapid Global Dissemination of the Clonal Group 258 Klebsiella pneumoniae Pandemic. PLoS One 10:e0133727.

74.	Klebsiella pneumoniae subsp. pneumoniae MGH 78578 genome assembly ASM1630v1. NCBI. https://www.ncbi.nlm.nih.gov/datasets/genome/GCA_000016305.1/. Retrieved 23 July 2026.

75.	Carrër A, Poirel L, Eraksoy H, Cagatay AA, Badur S, Nordmann P. 2008. Spread of OXA-48-positive carbapenem-resistant Klebsiella pneumoniae isolates in Istanbul, Turkey. Antimicrob Agents Chemother 52:2950–2954.

76.	Yong D, Toleman MA, Giske CG, Cho HS, Sundman K, Lee K, Walsh TR. 2009. Characterization of a new metallo-beta-lactamase gene, bla(NDM-1), and a novel erythromycin esterase gene carried on a unique genetic structure in Klebsiella pneumoniae sequence type 14 from India. Antimicrob Agents Chemother 53:5046–5054.

77.	Liao Y-C, Huang T-W, Chen F-C, Charusanti P, Hong JSJ, Chang H-Y, Tsai S-F, Palsson BO, Hsiung CA. 2011. An experimentally validated genome-scale metabolic reconstruction of Klebsiella pneumoniae MGH 78578, iYL1228. J Bacteriol 193:1710–1717.

78.	Liu Y-Y, Wang Y, Walsh TR, Yi L-X, Zhang R, Spencer J, Doi Y, Tian G, Dong B, Huang X, Yu L-F, Gu D, Ren H, Chen X, Lv L, He D, Zhou H, Liang Z, Liu J-H, Shen J. 2016. Emergence of plasmid-mediated colistin resistance mechanism MCR-1 in animals and human beings in China: a microbiological and molecular biological study. Lancet Infect Dis 16:161–168.

79.	Wyres KL, Hawkey J, Hetland MAK, Fostervold A, Wick RR, Judd LM, Hamidian M, Howden BP, Löhr IH, Holt KE. 2019. Emergence and rapid global dissemination of CTX-M-15-associated Klebsiella pneumoniae strain ST307. J Antimicrob Chemother 74:577–581.

80.	Norsigian CJ, Attia H, Szubin R, Yassin AS, Palsson BØ, Aziz RK, Monk JM. 2019. Comparative Genome-Scale Metabolic Modeling of Metallo-Beta-Lactamase–Producing Multidrug-Resistant Klebsiella pneumoniae Clinical Isolates. Front Cell Infect Microbiol 9.

81.	Cooper HB, Vezina B, Hawkey J, Passet V, López-Fernández S, Monk JM, Brisse S, Holt KE, Wyres KL. 2024. A validated pangenome-scale metabolic model for the Klebsiella pneumoniae species complex. Microbial Genomics 10:001206.

82.	Bergogne-Bérézin E, Towner KJ. 1996. Acinetobacter spp. as nosocomial pathogens: microbiological, clinical, and epidemiological features. Clin Microbiol Rev 9:148–165.

83.	Paton R, Miles RS, Hood J, Amyes SGB, Miles RS, Amyes SGB. 1993. ARI 1: β-lactamase-mediated imipenem resistance in *Acinetobacter baumannii*. International Journal of Antimicrobial Agents 2:81–87.

84.	Bouvet PJM, Grimont PAD. 1986. Taxonomy of the Genus Acinetobacter with the Recognition of Acinetobacter baumannii sp. nov., Acinetobacter haemolyticus sp. nov., Acinetobacter johnsonii sp. nov., and Acinetobacter junii sp. nov. and Emended Descriptions of Acinetobacter calcoaceticus and Acinetobacter lwoffii. International Journal of Systematic and Evolutionary Microbiology 36:228–240.

85.	Dijkshoorn L, Aucken H, Gerner-Smidt P, Janssen P, Kaufmann ME, Garaizar J, Ursing J, Pitt TL. 1996. Comparison of outbreak and nonoutbreak Acinetobacter baumannii strains by genotypic and phenotypic methods. J Clin Microbiol 34:1519–1525.

86.	Hamidian M, Nigro SJ. 2019. Emergence, molecular mechanisms and global spread of carbapenem-resistant Acinetobacter baumannii. Microb Genom 5:e000306.

87.	Vallenet D, Nordmann P, Barbe V, Poirel L, Mangenot S, Bataille E, Dossat C, Gas S, Kreimeyer A, Lenoble P, Oztas S, Poulain J, Segurens B, Robert C, Abergel C, Claverie J-M, Raoult D, Médigue C, Weissenbach J, Cruveiller S. 2008. Comparative Analysis of Acinetobacters: Three Genomes for Three Lifestyles. PLOS ONE 3:e1805.

88.	Kim HU, Kim TY, Lee SY. 2010. Genome-scale metabolic network analysis and drug targeting of multi-drug resistant pathogen Acinetobacter baumannii AYE. Mol BioSyst 6:339–348.

89.	Karthikeyan K, Thirunarayan MA, Krishnan P. 2010. Coexistence of blaOXA-23 with blaNDM-1 and armA in clinical isolates of Acinetobacter baumannii from India. J Antimicrob Chemother 65:2253–2254.

90.	Zumla A. 2010. Mandell, Douglas, and Bennett’s principles and practice of infectious diseases. Lancet Infect Dis 10:303–304.

91.	Livermore DM. 1992. Interplay of impermeability and chromosomal beta-lactamase activity in imipenem-resistant Pseudomonas aeruginosa. Antimicrob Agents Chemother 36:2046–2048.

92.	Walsh TR, Toleman MA, Poirel L, Nordmann P. 2005. Metallo-β-Lactamases: the Quiet before the Storm? Clinical Microbiology Reviews 18:306–325.

93.	Stover CK, Pham XQ, Erwin AL, Mizoguchi SD, Warrener P, Hickey MJ, Brinkman FS, Hufnagle WO, Kowalik DJ, Lagrou M, Garber RL, Goltry L, Tolentino E, Westbrock-Wadman S, Yuan Y, Brody LL, Coulter SN, Folger KR, Kas A, Larbig K, Lim R, Smith K, Spencer D, Wong GK, Wu Z, Paulsen IT, Reizer J, Saier MH, Hancock RE, Lory S, Olson MV. 2000. Complete genome sequence of Pseudomonas aeruginosa PAO1, an opportunistic pathogen. Nature 406:959–964.

94.	Oberhardt MA, Puchałka J, Fryer KE, Martins dos Santos VAP, Papin JA. 2008. Genome-scale metabolic network analysis of the opportunistic pathogen Pseudomonas aeruginosa PAO1. J Bacteriol 190:2790–2803.

95.	Jovcic B, Lepsanovic Z, Suljagic V, Rackov G, Begovic J, Topisirovic L, Kojic M. 2011. Emergence of NDM-1 metallo-β-lactamase in Pseudomonas aeruginosa clinical isolates from Serbia. Antimicrob Agents Chemother 55:3929–3931.

96.	Viedma E, Juan C, Villa J, Barrado L, Orellana MÁ, Sanz F, Otero JR, Oliver A, Chaves F. 2012. VIM-2–producing Multidrug-Resistant Pseudomonas aeruginosa ST175 Clone, Spain. Emerg Infect Dis 18:1235–1241.

97.	Focil C, Dalldorf C, Martinez D, Zepeda A, Zuniga C. 2026. Predicting strain-specific metabolic capabilities in the Genus Pseudomonas using a Flux-to-AI approach unravels hidden cell envelope properties. Computational and Structural Biotechnology Journal 0.

98.	Hormaeche E, Edwards PR. 1960. A Proposed Genus Enterobacter. International Bulletin of Bacteriological Nomenclature and Taxonomy 10:71–74.

99.	Sanders CC, Sanders WE. 1985. Microbial resistance to newer generation beta-lactam antibiotics: clinical and laboratory implications. J Infect Dis 151:399–406.

100.	Naas T, Nordmann P. 1994. Analysis of a carbapenem-hydrolyzing class A beta-lactamase from Enterobacter cloacae and of its LysR-type regulatory protein. Proc Natl Acad Sci U S A 91:7693–7697.

101.	Lee EH, Nicolas MH, Kitzis MD, Pialoux G, Collatz E, Gutmann L. 1991. Association of two resistance mechanisms in a clinical isolate of Enterobacter cloacae with high-level resistance to imipenem. Antimicrob Agents Chemother 35:1093–1098.

102.	Sanders WE, Sanders CC. 1997. Enterobacter spp.: pathogens poised to flourish at the turn of the century. Clin Microbiol Rev 10:220–241.

103.	de Champs C, Sirot D, Chanal C, Poupart MC, Dumas MP, Sirot J. 1991. Concomitant dissemination of three extended-spectrum beta-lactamases among different Enterobacteriaceae isolated in a French hospital. J Antimicrob Chemother 27:441–457.

104.	Bratu S, Landman D, Alam M, Tolentino E, Quale J. 2005. Detection of KPC carbapenem-hydrolyzing enzymes in Enterobacter spp. from Brooklyn, New York. Antimicrob Agents Chemother 49:776–778.

105.	Ren Y, Ren Y, Zhou Z, Guo X, Li Y, Feng L, Wang L. 2010. Complete genome sequence of Enterobacter cloacae subsp. cloacae type strain ATCC 13047. J Bacteriol 192:2463–2464.

106.	Chavda KD, Chen L, Fouts DE, Sutton G, Brinkac L, Jenkins SG, Bonomo RA, Adams MD, Kreiswirth BN. 2016. Comprehensive Genome Analysis of Carbapenemase-Producing Enterobacter spp.: New Insights into Phylogeny, Population Structure, and Resistance Mechanisms. mBio 7:e02093-16.

107.	Gürbüz M, Gencer G. 2024. Global trends and future directions on carbapenem-resistant Enterobacteriaceae (CRE) research: A comprehensive bibliometric analysis (2020-2024). Medicine (Baltimore) 103:e40783.
